# An Enhanced YOLOv8n-Based Method for Fire Detection in Complex Scenarios

**DOI:** 10.3390/s25175528

**Published:** 2025-09-05

**Authors:** Xuanyi Zhao, Minrui Yu, Jiaxing Xu, Peng Wu, Haotian Yuan

**Affiliations:** 1 School of Electronic Information and Electrical Engineering, Yangtze University, Jingzhou 434023, China; 2 School of Urban Construction, Yangtze University, Jingzhou 434023, China

**Keywords:** computer vision, object detection, image processing

## Abstract

With the escalating frequency of urban and forest fires driven by climate change, the development of intelligent and robust fire detection systems has become imperative for ensuring public safety and ecological protection. This paper presents a comprehensive multi-module fire detection framework based on visual computing, encompassing image enhancement and lightweight object detection. To address data scarcity and to enhance generalization, a projected generative adversarial network (Projected GAN) is employed to synthesize diverse and realistic fire scenarios under varying environmental conditions. For the detection module, an improved YOLOv8n architecture is proposed by integrating BiFormer Attention, Agent Attention, and CCC (Compact Channel Compression) modules, which collectively enhance detection accuracy and robustness under low visibility and dynamic disturbance conditions. Extensive experiments on both synthetic and real-world fire datasets demonstrated notable improvements in image restoration quality (achieving a PSNR up to 34.67 dB and an SSIM up to 0.968) and detection performance (mAP reaching 0.858), significantly outperforming the baseline. The proposed system offers a reliable and deployable solution for real-time fire monitoring and early warning in complex visual environments.

## 1. Introduction

In recent years, major fire accidents have occurred frequently worldwide, with their suddenness and destructive nature becoming increasingly prominent. As a cross-regional and multi-scenario disaster type, fires can occur in various environments, such as forests, grasslands, cities, and industrial areas, and exhibit common characteristics, such as rapid spread and difficulty to control. Some typical cases include the 2022 Australian Queensland wildfires, the 2023 Seoul city fires in South Korea, the 2024 Canadian forest fires, and the 2025 California mountain fires. These disaster events not only caused severe economic losses but posed multi-dimensional threats to human life, safety, social infrastructure, and natural ecosystems. It is worth noting that the continuous increase in the frequency of global fire accidents further highlights the necessity and urgency of strengthening research on fire prevention and control.

Over the past decade, research on fire detection systems has made significant progress. Conventional smoke detectors intrinsically lack the discriminative capacity to differentiate combustion-derived aerosols from benign particulate suspensions, which precipitates an elevated incidence of spurious alarms and, consequently, escalates operational and maintenance expenditures [1,2]. To transcend this inherent limitation, multi-modal detection paradigms have been rigorously devised that synergistically fuse optical sensors or RGB imagers with meticulously engineered descriptors encapsulating the full gamut of flame and smoke physical characteristics—chromatic signatures, spatio-temporal dynamics, spectral profiles, and fine-grained textural attributes—thereby endowing the detection framework with unprecedented discriminative capacity. Within this framework, colorimetric representations, including RGB [3,4], YCbCr [5], CIELAB [6], YUV [7,8], and HSV [9], have been systematically interrogated for early-stage fire recognition. Nonetheless, chrominance-based models persist in exhibiting prohibitively high false-positive rates, because univariate color information alone lacks the discriminative capacity requisite for robust and timely fire detection. In large-scale wildfire monitoring, satellite remote sensing [10,11] is intrinsically compromised by persistent cloud occlusion and the ubiquitous interference of non-wildfire thermal anomalies [12], whereas LiDAR systems [13,14] are critically debilitated by fog, haze, and other adverse atmospheric conditions; consequently, neither modality can deliver the real-time responsiveness nor the operational reliability demanded by early-warning applications. Image- and video-based detection modalities have consequently emerged as pivotal adjuncts, furnishing fire-identification frameworks with indispensable redundancy and enhanced fidelity. Sharma et al. [15] developed a fire detection system using VGG16 and Resnet50, improving accuracy on imbalanced datasets by adding fully connected layers, with Resnet50 performing slightly better, and facilitating more successful applications. Muhammad et al. [16] proposed a cost-effective CNN architecture inspired by GoogLeNet for monitoring video fire detection, balancing efficiency and accuracy, and verified its effectiveness on benchmark datasets after fine-tuning, suitable for closed-circuit television monitoring systems. Zhang et al. [17] proposed a deep learning method for forest fire detection, using a cascaded CNN classifier first to test the complete image for location and then to test the fire patch, achieving training and testing accuracy rates of 97% and 90%, respectively, establishing the first fire detection benchmark with patch-level annotations. Although these methods have considerable accuracy, they have the problem of a long detection delay time.

In recent years, image and video monitoring technologies based on deep learning have developed rapidly. Unlike traditional methods that rely on manually designed features, deep learning methods automatically learn and extract features from large datasets through neural networks, reducing human intervention and achieving more accurate and efficient object detection. Many researchers around the world have proposed various forest fire detection methods based on deep learning. Two-stage object detection algorithms, such as R-CNN, Mask R-CNN [18], and Faster R-CNN [19], provide high accuracy for fire detection. However, these algorithms have the drawbacks of high computational cost and slow inference speed, which makes them ill-suited to meet the requirements of real-time fire monitoring and difficult to be deployed on resource-constrained devices. By contrast, single-stage object detection algorithms, such as YOLO (You Only Look Once) [20] and SSD (Single Shot MultiBox Detector) [21], can directly perform object classification and regression without the need for additional region proposal steps. Therefore, they have a faster computing speed and inference efficiency, and are more suitable for real-time fire monitoring and deployment on resource-limited devices. Wu and Zhang [22] used SSD for fire detection and proposed a new structure, tiny-yolo-voc1, which improved the accuracy of fire detection. Seydi et al. [23] proposed a deep learning framework called Fire-Net, which integrates optical and thermal modalities from Landsat-8 images for detecting active wildfires and biomass burning. By using residual connections and depthwise separable convolutions, the model can extract more abstract and deeper features. Almeida et al. [24] improved the original EdgeFireSmoke algorithm and proposed EdgeFireSmoke++, which integrates an artificial neural network to effectively monitor forest fires through real-time video streams. Zhao et al. [25] proposed an improved deep learning algorithm, Fire-YOLO, based on YOLOv3. This model uses EfficientNet to extract features from the input image, enhancing feature learning. By expanding the feature extraction network in depth, width, and resolution dimensions, Fire-YOLO improves the recognition of small targets while reducing model parameters. However, it still faces problems, such as a low detection accuracy and difficulty in detecting partially occluded objects. Khan et al. [26] developed a cross-module attention network (CANet) for fire detection, optimizing the model by squeezing wide paths, channel attention, and multi-scale feature selection modules to improve detection accuracy and efficiency, and constructed a complex fire classification database. Wang et al. [27] proposed the YOLO-LFD model, optimizing YOLOv5 through depthwise separable convolutions, C2f-Light, and C3CIB, to improve inference speed and small fire detection accuracy, suitable for real-time fire detection on resource-constrained devices.

Despite a rapidly expanding body of research, the literature still lacks a fire-detection paradigm that concurrently reconciles visual scene complexity, state-of-the-art accuracy, and ultra-low latency. To redress this deficit, we introduce an end-to-end vision-intelligent solution and design a pioneering early-warning framework that coherently integrates multi-modal image analytics with a deep attention mechanism. The principal contributions of this study are articulated as follows:

1. A multi-stage image-enhancement pipeline that integrates denoising, dehazing, and deblurring to guarantee high-fidelity inputs under adverse visual conditions;
2. A data-centric strategy leveraging Projected GAN for high-diversity fire-scene augmentation, substantially improving model generalizability;
3. A compact yet potent YOLOv8n-based detector that embeds the Bi-Former Attention, Agent Attention, and the CCC units to sustain robust performance in low-visibility and highly dynamic environments.

## 2. System Theoretical Foundation

**Figure 1:**
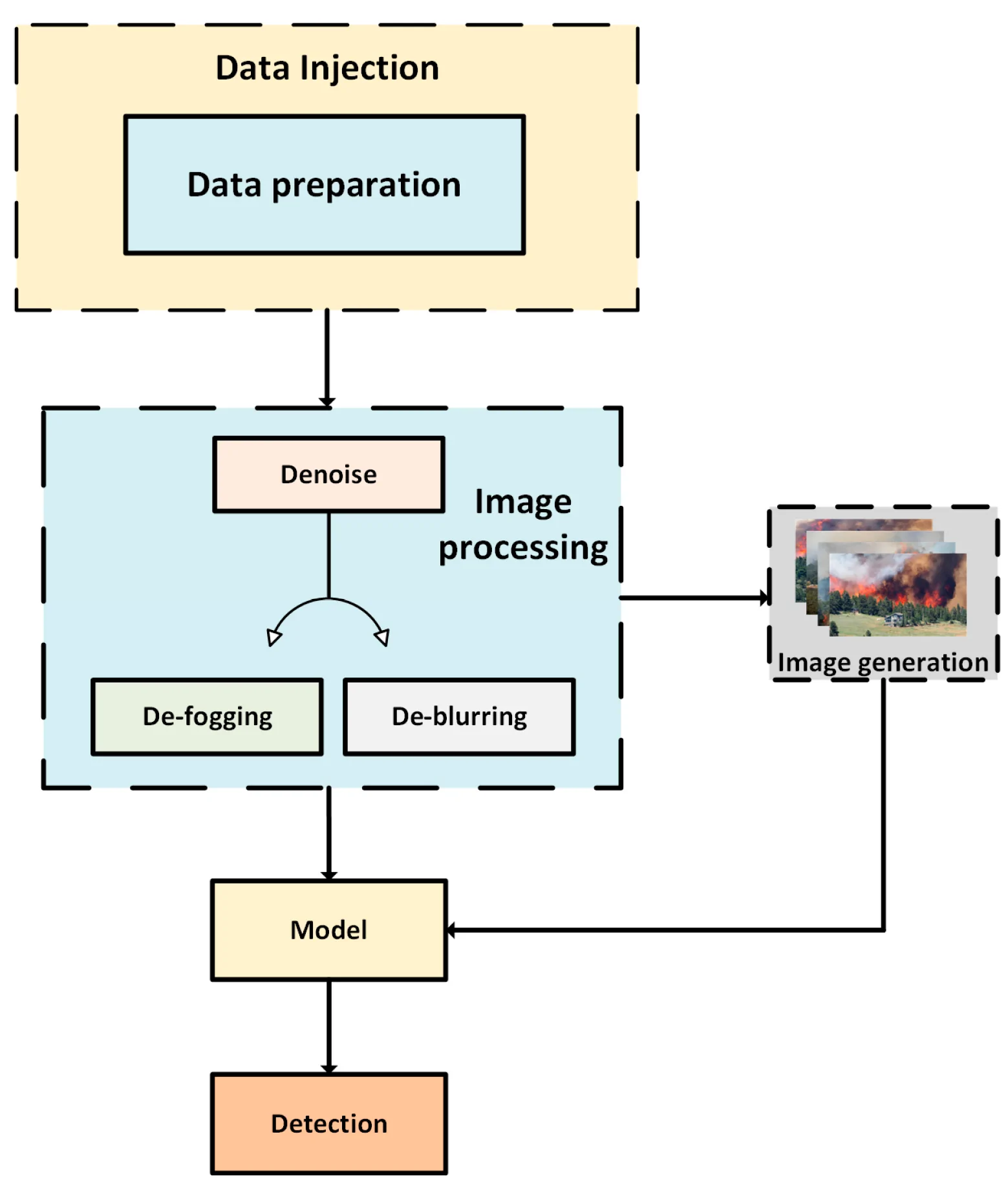
Flowchart of Machine Vision-Based Fire Detection in Complex Environments.

To construct a fire visual warning system with high robustness, strong generalization, and secure controllability, this section analyzes the core technical components from a functional perspective and outlines the theoretical underpinnings of the system architecture, as illustrated in Figure 1.

At the data injection stage, all incoming images are routed to the data preparation module, where uniform standardization is applied in terms of resolution, format, and channel configuration, ensuring consistency across subsequent processing stages.

Once sanitized, the images proceed to the core image processing module, which comprises three key enhancement sub-tasks: denoising, dehazing, and deblurring. These tasks operate in an integrated fashion to correct typical degradation phenomena in fire imagery, including sensor noise, atmospheric scattering, and motion-induced blur. Notably, the denoising network employed in this framework leverages group sparse constraints and an attention-guided mechanism, enabling it to suppress background noise while preserving critical texture and structural details.

Beyond pre-processing, the system offers an image generation branch that utilizes the processed images to augment the training dataset, further boosting the model’s generalization performance in data-scarce scenarios. Finally, all processed and enhanced images are passed into the downstream fire detection model, where real-time and reliable identification of fire-related features is carried out.

### 2.1. Image Denoising

The literature on image denoising covers a variety of methods aimed at effectively reducing noise while preserving key image details (such as edges and textures). Li et al. [28] proposed a method that combines soft thresholding processing with edge enhancement techniques. Their approach utilizes the Canny edge detection operator to identify image features, and then pre-processes the image through a flat operator. Subsequently, the denoised edge image and the noisy image are subjected to stationary wavelet transform, and the wavelet coefficients are combined at corresponding levels. This process facilitates soft threshold denoising and effectively suppresses noise while maintaining the integrity of the edges. Based on the transform-based technique, Chen and Zhou [29] proposed using contour wavelet transform for image denoising. Their research results showed that this method outperforms traditional wavelet-based methods in terms of signal-to-noise ratio (SNR), image enhancement factor (IEF), and visual quality, indicating its superior ability in capturing image features and reducing noise. Besides transform-domain methods, filtering techniques, such as bilateral filters, have also been studied due to their denoising performance [30]. This study explored how the efficacy of filters is affected by parameters such as window size, spatial variance, and radiation variance. This research emphasized the importance of adjusting parameters in bilateral filtering to optimize noise reduction without compromising image details. The reduction of speckle noise, especially in medical imaging, has been addressed through advanced minimization techniques. Thapa et al. [31] proposed a multi-frame weighted nuclear norm minimization (MWNNM) method for spectral domain optical coherence tomography (SD-OCT) images. Their method extends the weighted nuclear norm minimization framework to multiple frames, achieving more effective speckle noise suppression compared to single-frame methods. In addition to traditional filtering and transformation methods, recent developments have included methods based on deep learning. Majumdar [32] proposed a blind denoising autoencoder trained directly on noisy samples, marking a new breakthrough in denoising methods based on autoencoders. This method can learn denoising without prior knowledge of noise features, representing a significant advancement in unsupervised denoising techniques. Moreover, the decomposition-based framework has been used in compressive sensing reconstruction research. Devi and Patil [33] evaluated various filtering techniques for microscope images, emphasizing the importance of adopting customized methods for different imaging modalities. Yuan et al. [34] addressed sonar image denoising by analyzing noise features and using a gamma distribution-based expert domain (FoE) model, demonstrating the effectiveness of combining statistical analysis with learning priors in complex noise scenarios.

In the image denoising task, especially when dealing with real-world noisy images, traditional degradation assumptions based on Gaussian distribution modeling often struggle to accurately capture the complex noise structure. To address this issue, this paper proposes a novel blind image denoising network that combines group sparsity constraints with an attention-guided transformer (AGT). This approach takes into account both structural priors and long-range dependency modeling capabilities, achieving excellent results.

The entire denoising model consists of two key components: a Group Sparse Denoising Network (GSDNet) and an Attention-Guided Transformer Module (AGT). The GSDNet employs a multi-scale CNN structure and incorporates group sparsity regularization to initially suppress background noise and to retain local texture information. The AGT module further exploits the distant context relationships through a carefully designed window attention mechanism, enabling detail restoration.

In the group sparsity constraint, the network encodes image feature blocks and constructs a group sparse structure in the feature channel dimension. This method leverages the coefficient sparsity of natural images in the frequency domain or spatial domain, effectively suppressing unstructured noise. The mathematical modeling is as follows:

(1) $$\underset{X}{min}\;∥Y-X{∥}_{2}^{2}+\lambda \sum_{g=1}^{G}\;∥X_{g}{∥}_{2}$$

Here, Y represents the noisy image, X represents the denoised image, $X_{g}$ denotes the feature representation of the g-th channel group, and λ is the sparsity constraint coefficient. This formula retains the local structure while forcing the feature blocks to be sparsely distributed within the group, effectively eliminating non-structural components.

Secondly, the attention-guided transformer module enhances the information flow ability through cross-window interaction. The overall network structure adopts the residual connection form and is composed of multiple AGT blocks stacked together. Each AGT block contains multi-head attention layers and a feedforward neural network (FFN). The attention mechanism can be expressed as follows:

(2) $$Attention(Q,K,V)=softmax\left(\frac{QK^{T}}{\sqrt{d_{k}}}\right)V$$

Here, Q, K, and V represent the input query, key, and value vectors, respectively, and $d_{k}$ is the dimension of the key vector. In practical applications, the transformer module employs a sliding window attention mechanism, thereby maintaining computational efficiency while capturing long-range dependencies.

**Figure 2:**
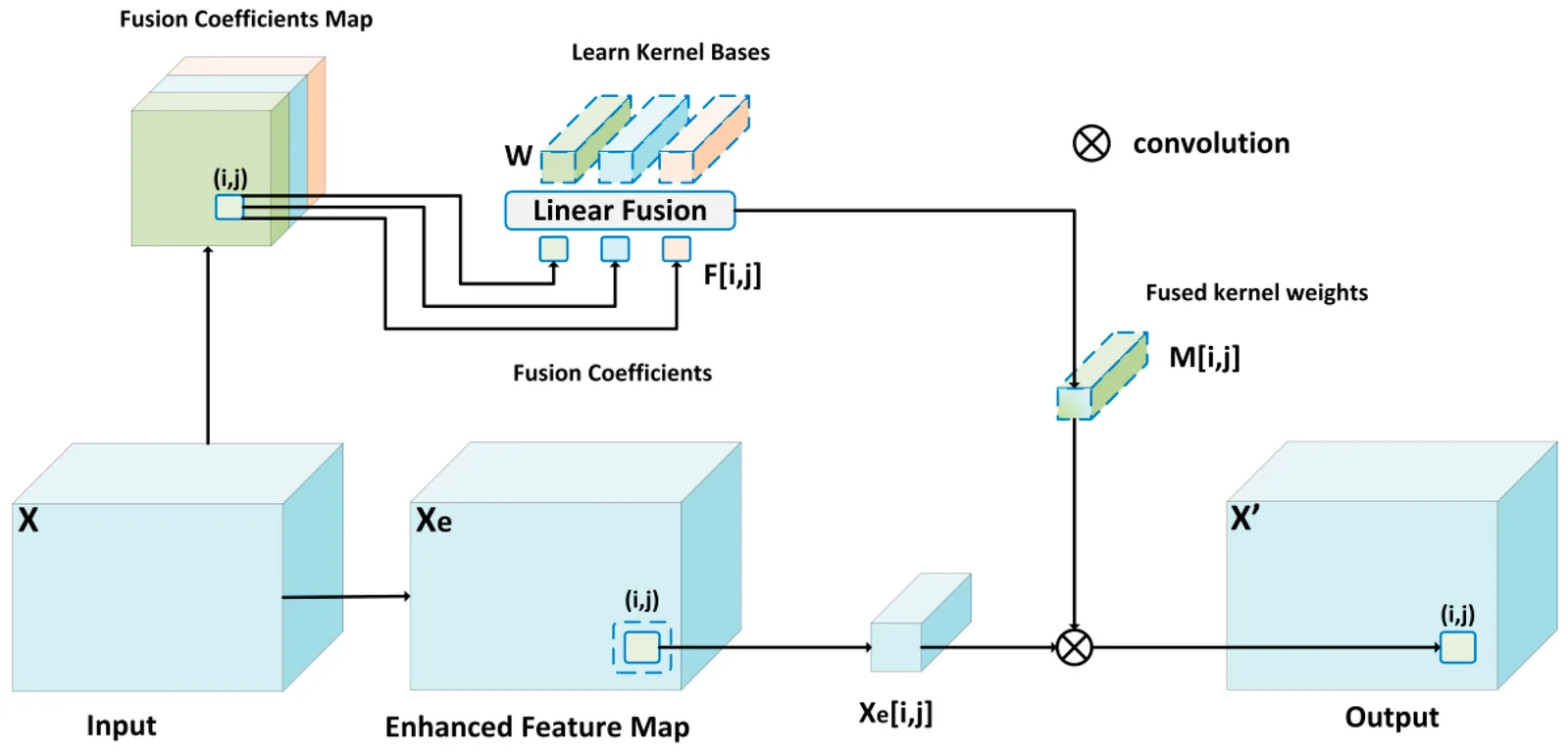
Architecture of the Dynamic Kernel Fusion Convolution Module.

As shown in Figure 2, a network with a dynamic kernel fusion convolution structure is introduced. The core idea is to adaptively generate convolution kernel parameters at specific positions to enhance the spatial context modeling ability and to improve the network’s expression ability in image denoising, image super-resolution, or image enhancement tasks. This structure consists of three main sub-modules: feature enhancement branch, fusion coefficient generator, and dynamic convolution kernel generator.

The input image X ∈ R, H × W × C is extracted as the initial feature map Xe through convolution. At each position (i, j), the network predicts the fusion coefficient F[i, j] for that position using an independent coefficient mapping network, which is used to weight K predefined convolution kernel bases W1, W2, …, WK.

In addition, this method also introduces a residual enhancement module and feature fusion strategy to avoid problems such as image smoothing and texture loss, thereby improving the final image quality. This framework effectively overcomes the limitations of traditional methods in noise modeling and context perception by combining the group sparsity constraints and the attention mechanism, providing a feasible and efficient blind image denoising solution.

### 2.2. Image Blurring Modeling and Restoration

In actual fire smoke scenarios, blurring phenomena occur widely at all stages of image acquisition, such as camera jitter, smoke obstruction, and image quality degradation under low illumination conditions, which seriously affect the response efficiency and accuracy of subsequent fire situation recognition and smoke warning systems. To effectively restore the clarity of images and to enhance the perceptual representation of fire-region features, researchers have proposed various methods around single-image dehazing and deblurring technologies. In dehazing, Sun et al. proposed the semi-supervised single-image dehazing network SADnet based on the attention mechanism, which can effectively guide the model to learn dehazing features in the case of insufficient supervision, enhancing the expression ability of flames and smoke boundaries in the image [35]. Liang et al. designed a heterogeneous prior-driven dehazing method for remote sensing images, which is suitable for smoke removal in complex environments and has good generalization performance [36]. Liao et al. proposed an image dehazing model without supervision based on fuzzy clustering and local structure information, suitable for application in scenarios with limited imaging in initial fires [37]. In addition, Guo et al. and Liu et al. systematically reviewed the application of image dehazing methods in information fusion and remote sensing image processing from a technical review perspective, providing theoretical support for fire image enhancement [38,39]. In the field of image deblurring, Yang and Evans proposed lightweight deblurring methods for resource-constrained platforms, suitable for embedded fire monitoring equipment [40]. Xu and Wei designed an unsupervised deblurring method with a pyramid structure based on deep image priors, avoiding the reliance on real clear images and having good application promotion capabilities [41]. Li et al. integrated polarization information and improved underwater dehazing performance through multi-index reconstruction strategies, whose idea can also be transferred to fire images with heavy smoke [42]. At the same time, the transformer structure has demonstrated strong modeling capabilities in the deblurring task. For example, Tsai et al. proposed Stripformer, which improved the image restoration speed through strip-shaped feature modeling [43], and Kong et al. used a frequency-domain transformer to enhance the clarity performance of blurred images [44]. Moreover, Dong et al. proposed the multi-scale residual filtering network [45], and Ren et al. introduced the structure-guided diffusion modeling method [46], achieving excellent results in complex blurring conditions. Ji et al. and Kim et al., respectively, adopted the “divide and conquer” and multi-stage structure to perform efficient deblurring on a single image, providing a solution with both structure and performance for the dynamic image blurring processing of fire images [47,48].

**Figure 3:**
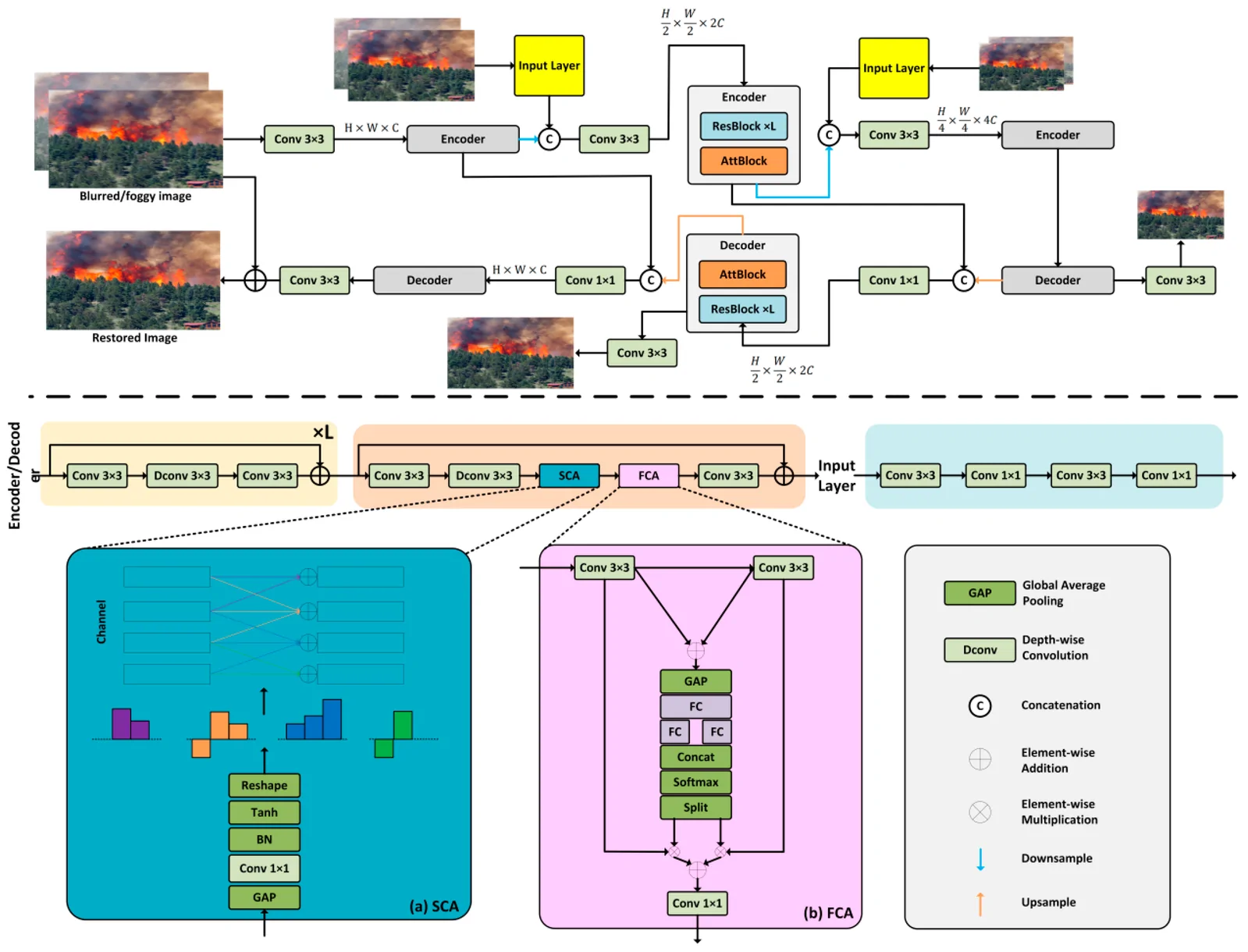
The ChaIR Network Structure for Blur Restoration.

Specifically, such blurring can be classified into two main categories: static blurring and dynamic blurring. The former is mainly caused by the degradation of the image signal itself, such as sensor noise, focal length deviation and compression loss; the latter stems from temporal sequence-based dynamic changes, such as target movement, camera jitter, or thermal air disturbance. Due to the fact that fire scenes often exist in complex lighting and motion interaction environments, a single image enhancement method is difficult to handle the modeling and restoration of various types of blurring. Therefore, this paper designs a two-stage blurring modeling framework, combining static noise suppression and dynamic blurring correction strategies, to uniformly complete the multimodal blurring restoration process within the ChaIR network architecture. This module provides a clearer and more robust image foundation for subsequent target detection. As shown in Figure 3, this network adopts an encoder–decoder architecture, integrating the spatial-channel attention module (SCA) and the frequency-channel attention module (FCA), respectively, to model the static and dynamic blurring features. Each stage achieves feature fusion through multi-scale residual connections and introduces explicit frequency-domain modulation to enhance the high-frequency restoration capability.

To quantitatively evaluate the restoration effect of the image deblurring module, this paper introduces two image quality evaluation indicators: the structural similarity index (SSIM) and the peak signal-to-noise ratio (PSNR). The SSIM mainly measures the fidelity of the image in terms of brightness, contrast, and structure. Its calculation formula is as follows:

(3) $$SSIM(x,y)=\frac{\left(2\mu_{x}\mu_{y}+C_{1})(2\sigma_{xy}+C_{2}\right)}{\left(\mu_{x}^{2}+\mu_{y}^{2}+C_{1})(\sigma_{x}^{2}+\sigma_{y}^{2}+C_{2}\right)}$$

Here, $\mu_{x}$ and $\mu_{y}$ represent the mean values of image x and y, respectively, $\sigma_{x}^{2}$ and $\sigma_{y}^{2}$ are the variances, $\sigma_{xy}$ is the covariance, and $C_{1}\;$ and $C_{2}$ are stability constants. When the SSIM value is closer to 1, the closer the image is to the real image.

The PSNR measures the image reconstruction error at the pixel level and is defined as follows:

(4) $$PSNR=10\cdot {log}_{10}(\frac{L^{2}}{MSE})$$

Here, L represents the maximum possible value of the image pixel (for an 8-bit image, it is 255), and MSE represents the image mean square error, which is defined as follows:

(5) $$MSE=\frac{1}{mn}\sum_{i=1}^{m}\;\sum_{j=1}^{n}\;[I(i,j)-K(i,j){]}^{2}$$

Here, I(i,j) and K(i,j) represent the pixel values at position (i,j) in the original image and the restored image, respectively, with m × n being the image size. The smaller the MSE, the less the image distortion, and the larger the PSNR, the better the restoration effect.

In the fuzzy modeling and deblurring module constructed in this paper, by comparing the PSNR and SSIM values between the restored image and the clear image, the model’s adaptability to static and dynamic blurry scenes can be effectively measured. The experimental results show that, when dealing with smoke obstruction, this method demonstrates superior performance compared to traditional approaches in both indicators, providing a more reliable input quality guarantee for subsequent intelligent defect detection and image recognition.

#### 2.2.1. Data Preparation

**Figure 4:**
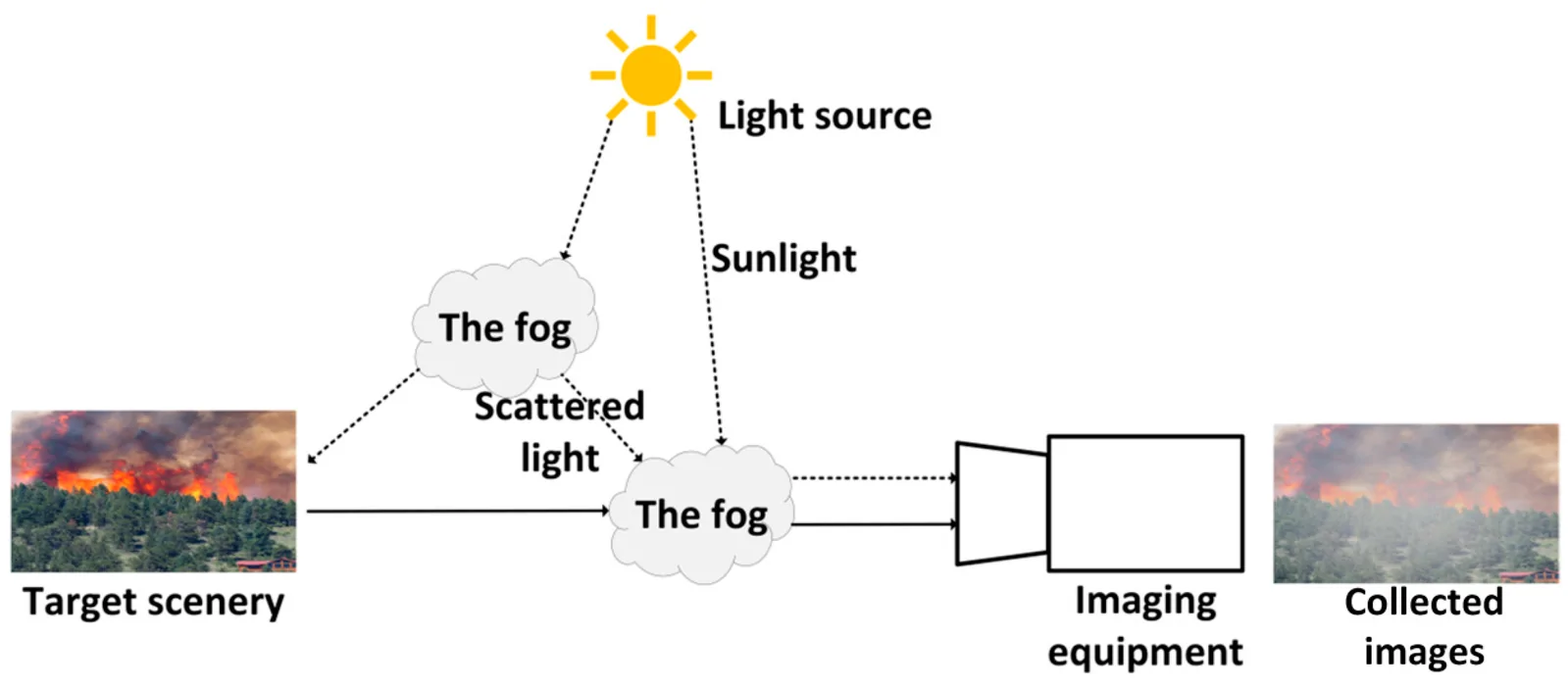
Illustration of the Physical Mechanism Behind Fog Image Generation.

In order to simulate the image degradation process affected by haze in a natural environment and to construct a reliable training dataset, this paper adopts an image synthesis method based on the atmospheric scattering model to simulate the presence of haze in clear images. Figure 4 illustrates the physical mechanism of light propagation in the haze environment during natural imaging. Under typical atmospheric conditions, the radiation signals from the target scene will be subject to the dual effects of scattering and absorption by water vapor particles (haze) before reaching the imaging device, ultimately resulting in phenomena such as decreased contrast, color shift, and structural blurring in the imaging image.

This process can be formally described by the classic atmospheric scattering model (ASM) as follows:

(6) I(x)=J(x)⋅t(x)+A⋅(1−t(x))

Here, I(x) represents the foggy image that is finally observed, J(x) is the original clear image, A is the global atmospheric light value, and t(x) is the transmittance function, which is defined as follows:

(7) $$t(x)=e^{-\beta \cdot d(x)}$$

Here, β represents the scattering coefficient of fog particles in the atmosphere and d is the physical depth information at pixel x. The above formula indicates that the fog’s shading effect is in an exponential decay relationship with the relative depth of the target from the camera.

As shown in Figure 4, the image obtained by the imaging device not only contains the direct light component emitted by the target scene (reduced by transmittance t(x)), but includes additional light intensity introduced by atmospheric light A and the scattering path. This scattered light significantly increases with the increase in fog concentration, resulting in the loss of local details in the image. By adjusting the parameters β, A, and the depth map d(x), the fog density and its spatial distribution can be flexibly controlled, generating diverse degraded image data.

This synthesis method provides sufficient labeled data for the subsequent training of the image dehazing network, avoiding the high cost and difficult labeling problems encountered in the real acquisition of haze images. At the same time, it also supports the combination of real depth maps or simulated depth scenarios to construct more accurate synthetic fog images, enabling more robust, weakly supervised and unsupervised learning for image enhancement.

Meanwhile, during the actual image acquisition process, due to the high-speed movement of the target object, the jitter of the camera platform, and the excessively long exposure time, it is very easy to cause the target edges in the image to have trailing and stretching phenomena, forming what is called dynamic motion blur images. This type of blur has stronger spatial non-uniformity compared to static blur, and its blur kernel changes with the different spatial positions of the image, seriously affecting the image clarity and the robustness of subsequent visual tasks.

The physical imaging process of dynamic blur can be formally expressed as follows:

(8) $$B(x)=\int_{0}^{T}\;J(x+v(x,t)\cdot t)dt+n(x)$$

Here, B(x) represents the blurred image, J(x) represents the clear image, v(x,t) represents the velocity vector of the pixel position x at time t, T is the exposure duration, and n(x) is the sensor noise. This integral form indicates that the blurred image is formed by the cumulative superposition of multiple instantaneous images of the target at different time positions.

This paper adopts an image degradation method based on the superposition of linear motion kernels: according to the preset velocity field or displacement trajectory, a set of intermediate images in consecutive time frames are generated; then, through temporal mean or weighted accumulation, an equivalent blurred image to the real acquisition system is obtained. This method not only can simulate the linear blurring caused by uniform translational motion, but can extend to support nonlinear displacement, rotational blurring, camera zooming and other complex situations.

It is worth noting that, to improve the authenticity of the synthesis of the blurred image, this paper introduces random jitter vectors, kernel distortion, scene occlusion, and foreground crossing, which are possible perturbation factors in real shooting processes, into the image generation process, thereby constructing a high-fidelity and diverse dynamic blurred image dataset, providing effective training support for subsequent deep deblurring models. Through this simulation mechanism, not only is the generalization ability of the training data improved, but a unified benchmark is provided for the evaluation of various deblurring algorithms in real complex scenarios.

#### 2.2.2. Image Dehazing

In the scenarios of fire and smoke warning, image dehazing serves as a crucial pre-processing step, which is of great significance for enhancing the accuracy and robustness of subsequent detection models. Due to the extensive dispersion of smoke during the initial stage or the spread process of a fire, significant phenomena, such as low contrast, color deviation, and blurred edges, will occur in the image. These degradations will seriously interfere with the model’s ability to recognize flames, smoke contours, and background structures. Therefore, effectively removing the interference of haze and restoring the intrinsic structure of the image is one of the core links in improving the performance of the visual perception system.

This paper adopts a deep learning-driven end-to-end image dehazing method. Without relying on the real transmittance and depth map, it achieves global structure restoration and detail enhancement of the haze map. The ChaIR network is used as a unified image restoration framework. In this task, through a specialized data preparation strategy and target optimization, it guides its learning of the dehazing transformation relationship.

In the network design, the ChaIR network enhances the decoupling and processing capabilities of the model for low-frequency (background haze layer) and high-frequency (edge details) information by introducing a frequency channel attention module (FCA). Compared to traditional CNNs, the FCA module can explicitly enhance the remaining structural texture signals during reconstruction, especially in the transition areas between thin smoke and atmospheric diffusion, showing higher reconstruction quality. To further optimize the training process, this paper introduces a dual-domain loss function as follows:

(9) $$L=L_{s}+\lambda \cdot L_{f}$$

Here, $L_{s}$ represents the pixel space $L_{1}$ loss, while $L_{f}$ represents the frequency domain $L_{1}$ loss, which is used to enhance the model’s robustness under changes in illumination distribution.

#### 2.2.3. Image Deblurring

In the actual deployment of the fire monitoring system, image blurring is also a problem it faces. This problem includes both static blurring caused by the image acquisition device (such as incorrect focal length, compression artifacts, etc.) and dynamic blurring caused by camera jitter, target movement, or thermal disturbances. Such blurring seriously affects the accuracy of identifying key features, such as flame edge contours and smoke diffusion trajectories. Therefore, in order to improve the spatial recognition and the structural clarity of the image, it is necessary to introduce a deblurring mechanism with dual-domain modeling capabilities to uniformly restore both the static and dynamic blurring features.

Research on image dehazing for single images has made significant progress, covering a variety of methods from traditional model-based approaches to advanced deep learning architectures. Early studies, such as the study by Matlin and Milanfar [49], mainly focused on methods that could simultaneously remove fog and noise from a single image, emphasizing the challenge of handling multiple degradation problems without relying on multiple images. This method highlights the importance of developing single-image solutions, especially in cases where multiple shots cannot be taken. On this basis, Fang et al. [50] proposed a fast variational method, which uses an adaptive window method based on the dark channel prior to estimate the transmission map, thereby achieving efficient dehazing and denoising simultaneously. This method reflects the trend of using prior-based models to improve dehazing performance. Further progress includes the research results of Shin et al. [51], who used convolutional network architecture to estimate environmental light and transmission maps, especially in underwater images. Their method improves the reconstruction quality by jointly estimating these parameters, addressing the unique challenges of the underwater environment. Perez et al. [52] demonstrated that deep learning techniques can generate high-quality image restoration effects from a single foggy image, inspired by successful cases in related image processing tasks (such as colorization and object detection). Based on this paradigm, Yang and Sun [53] proposed Proximal Dehaze-Net, which learns dark channel and transmission prior knowledge by expanding the iterative algorithm into a deep network, integrating prior knowledge into a learnable framework. Similarly, Zhang et al. [54] introduced a dehazing method for sky and river scenes, using external and internal cues to improve the dehazing effect in these specific scenarios.

The defuzzification strategy adopted in this paper is based on the ChaIR (Channel Interaction Restoration) network structure. This structure fully exploits the feature differences of image blurring degradation in the channel dimension. Through the spatial domain channel attention module (SCA) and the frequency domain channel attention module (FCA), it realizes the reconstruction of the blurred components and the restoration of details. The ChaIR structure adopts a U-shaped main network and combines multiple residual blocks to construct a feature extraction and reconstruction path. At the end, a channel attention mechanism is introduced to effectively enhance the dynamic selection ability of fuzzy information between feature layers.

The SCA module aims to conduct neighborhood interaction and weighted integration of each channel in the convolutional feature map, thereby enhancing the structural information of the blurred area and suppressing redundant background signals. Its specific calculation form is as follows:

(10) $$W=tanh\left(BN(f_{1\times 1}(GAP(I)))\right)\in R^{kC\times 1\times 1}$$

(11) $$I_{c}=\sum_{j=1}^{k}\;W_{c,j}\cdot I_{c+offset(c,j)}\in R^{H\times W}$$

Here, I $\in R^{H\times W}$ represents the input feature map, GAP is the global average pooling, $f_{1\times 1}$ represents the $f_{1\times 1}$ convolution, BN represents batch normalization, and the tanh activation function is used to generate positive and negative weighting factors to enhance the information filtering ability. Compared to the traditional Softmax attention, the SCA module can generate negative weights and suppress useless or ambiguous channel features.

In dynamic blurred images, key information is often reflected in the high-frequency part of the image. Therefore, the FCA module expands the expression ability of high-frequency information through multiple convolution paths and performs frequency fusion based on channel weights. The core calculation process is as follows:

(12) $$F_{1}=f_{3\times 3}^{1}(I),F_{2}=f_{3\times 3}^{2}(F_{1})$$

(13) $$W_{i}={FC}_{i}({FC}_{0}(GAP(\sum_{i}\;F_{i}))),i\in \{1,2\}$$

(14) $$W_{c}^{’}=\frac{e^{[W_{1},W_{2}{]}_{c}}}{\sum_{j=1}^{2C}\;e^{[W_{1},W_{2}{]}_{j}}}$$

(15) $$\hat{I}=f_{1\times 1}(F_{1}\cdot W_{1}^{’}+F_{2}\cdot W_{2}^{’})$$

During the above process, multiple frequency branches $F_{i}$ capture multi-frequency information in the blurry image through different-depth convolutions, and then use the sequential addition and Softmax calculation to obtain the fusion weights $W_{1}^{’}$. Subsequently, channel-weighted superposition is performed to restore the clear structure of the image.

### 2.3. Image Data Augmentation

In industrial visual inspection tasks, the scarcity of data and the imbalance of defect samples have long constrained the generalization ability of deep learning models. This paper selects Projected GAN as the main method to improve the quality and diversity of image generation. Projected GAN effectively avoids the problems of discriminator overfitting and instability in small sample training in the previous StyleGAN series of models by introducing a multi-scale feature space discriminator and combining the discriminative signals of the traditional image space.

The network architecture mainly includes two innovations: Firstly, the discriminator no longer directly classifies the image as true or false, but uses a fixed pre-trained feature extraction network to map the image to the semantic feature space and to make judgments at multiple scales, thereby improving the discriminative ability for structural information and local details. Secondly, the projection mechanism ensures that the generator obtains more explicit and stable gradient feedback during training, effectively improving the training stability and the fidelity of the final generated image.

**Figure 5:**
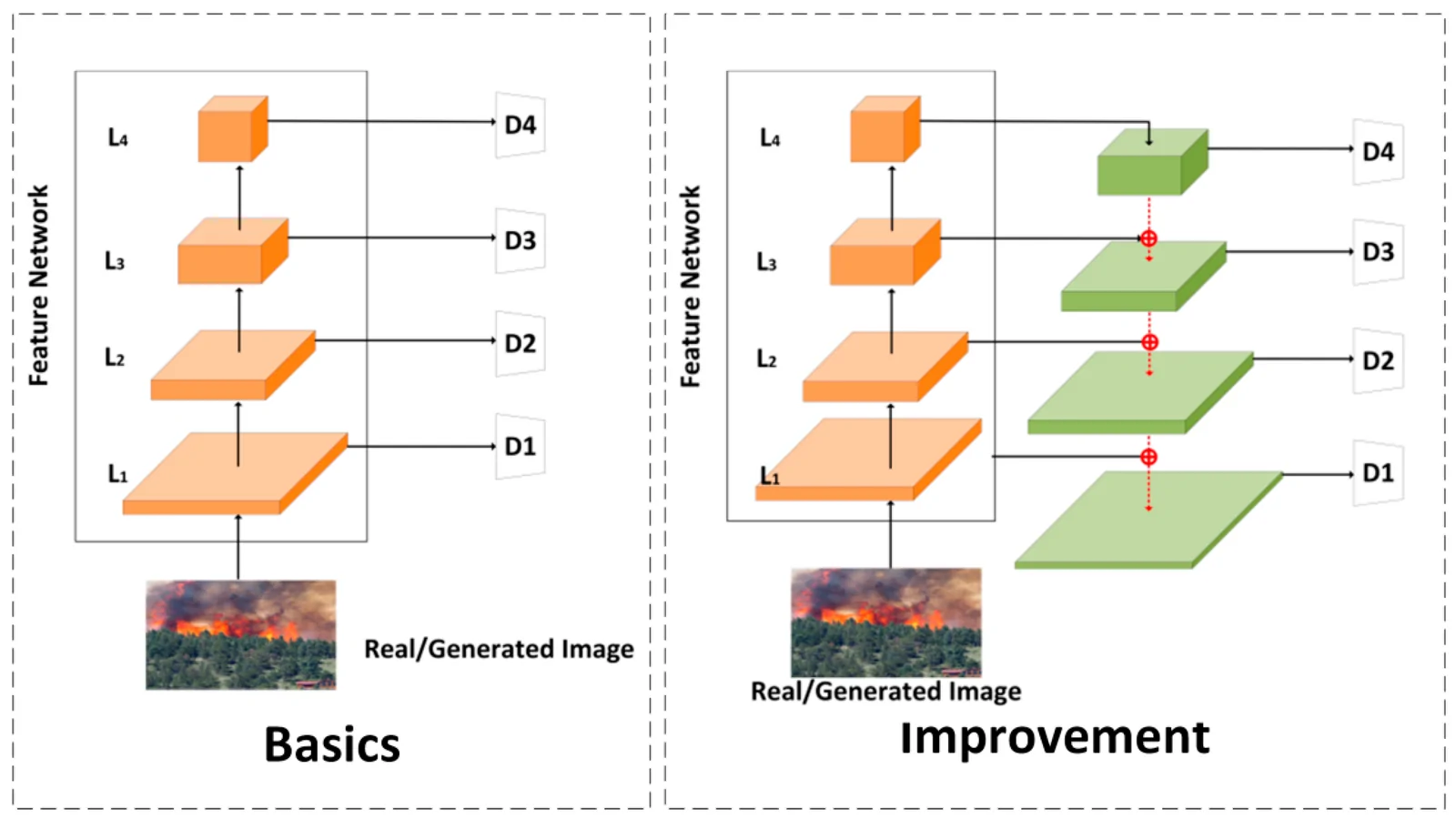
Projected GAN Network Structure.

The structure of the Projected GAN network is shown in Figure 5. In the basic structure, the discriminator first performs the multi-level feature encoding of the input image through the main feature extraction network. Specifically, the image is sent layer by layer to four different convolution modules from L1 to L4, corresponding to different spatial resolutions and semantic depths. These intermediate features are then sent to the D1 to D4 discriminator heads to perform true/false discrimination for each layer’s feature representation. This design enhances the collaborative modeling ability of local and global structures, enabling the discriminator to more accurately capture the texture details and semantic consistency of the image, thereby effectively improving the learning efficiency of the generator.

Based on this, Projected GAN introduces an auxiliary projection structure in the perceptual space. At each scale of the feature hierarchy, the discriminator not only uses the convolutional features from the original image, but aligns them with the perceptual features extracted by a fixed pre-trained network. The green feature branch in Figure 5 represents this projection path. Through the fusion between the perceptual space and the original feature space, the model obtains higher level semantic consistency constraints in the discrimination stage. This alignment mechanism prompts the generator not only to imitate real images at the pixel level, but to maintain the consistency of texture structure and style in the perceptual dimension.

This design significantly enhances the discrimination power of the discriminator and the convergence quality of the generator. Multi-scale supervision enhances the model’s sensitivity to the different scale details of the image, while the perceptual space alignment effectively avoids the blurring and structural drift problems of the generated images in complex backgrounds or weak texture areas.

Projected GAN in this system not only enhances the diversity and generalization ability of the training data, but provides controllable and high-fidelity sample support for the defect detection network under conditions of scarce data, promoting the intelligent development of industrial vision systems.

To further quantitatively evaluate the performance of the generated images of Projected GAN adopted in this paper, in terms of structural fidelity and semantic consistency, this paper introduces the Fréchet Inception Distance (FID) as an unrefereed image generation quality evaluation indicator. The FID is an authoritative measurement method widely used in the field of image generation and adversarial networks at present. Its core idea is to compare the statistical distributions of the generated images and the real images in the feature space, thereby evaluating the differences between the two.

Specifically, the FID assumes that images follow a multivariate Gaussian distribution in the feature space. Let the distribution of the features of the real images be N($\mu_{r}$, $\Sigma_{r}$), and the distribution of the features of the generated images be N($\mu_{g}$, $\Sigma_{g}$). Then, the Fréchet distance between the two is defined as follows:

(16) $$FID=∥\mu_{r}-\mu_{g}{∥}^{2}+Tr\left(\Sigma_{r}+\Sigma_{g}-2{\left(\Sigma_{r}\Sigma_{g}\right)}^{1/2}\right)$$

The smaller the FID value, the closer the generated image is to the real image in terms of perceptual quality and semantic structure, that is, the better the generation effect. Different from traditional pixel-level indicators, such as the PSNR and SSIM, the FID measures the statistical distribution difference of the image in the high-level feature space. Therefore, it is more sensitive to image content, style, and semantic integrity, and is suitable for the authentic evaluation of complex image structures, such as flame shapes and texture edges.

Research in the field of object detection has made significant progress due to the application of deep learning methods. Sun et al. [55] provided an overview of the evolution of object detection methods, highlighting the process from traditional approaches to complex deep learning models. These developments have made it possible to achieve more accurate and reliable detection in various scenarios. Recent studies have emphasized the importance of multimodal data fusion for improving detection performance. Open set target detection has also attracted attention. Liu et al. [56] developed Grounding DINO, a detector based on transformer, which identifies arbitrary objects by integrating pre-training based on human-provided category names or expression representations. Similarly, Wu et al. [57] introduced GRiT, a generative transformer, which can understand open set targets without predefined object categories, expanding the detection range and making it no longer limited to fixed categories. In response to the challenges brought by visual degradation scenarios, Liu et al. [58] proposed a guided detection method based on image enhancement. This method integrates an enhancement branch in an end-to-end manner in the detection network, aiming to improve detection performance in challenging visual environments. The application of object detection to specific domains, such as synthetic aperture radar (SAR) images, has also been explored. Li et al. [59] proposed a large-scale SAR dataset named SARDet-100K and a multi-stage (MSFA) pre-training framework with filter enhancement. This framework addresses the domain gap between RGB and SAR data, facilitating better transfer learning and detection performance in SAR environments. In summary, the literature reflects the expansion of object detection research, including multimodal fusion, robustness under environmental changes, open set recognition, and challenges in specific domains, which is driven by innovative architectures and training strategies by Jiang et al. [60].

**Figure 6:**
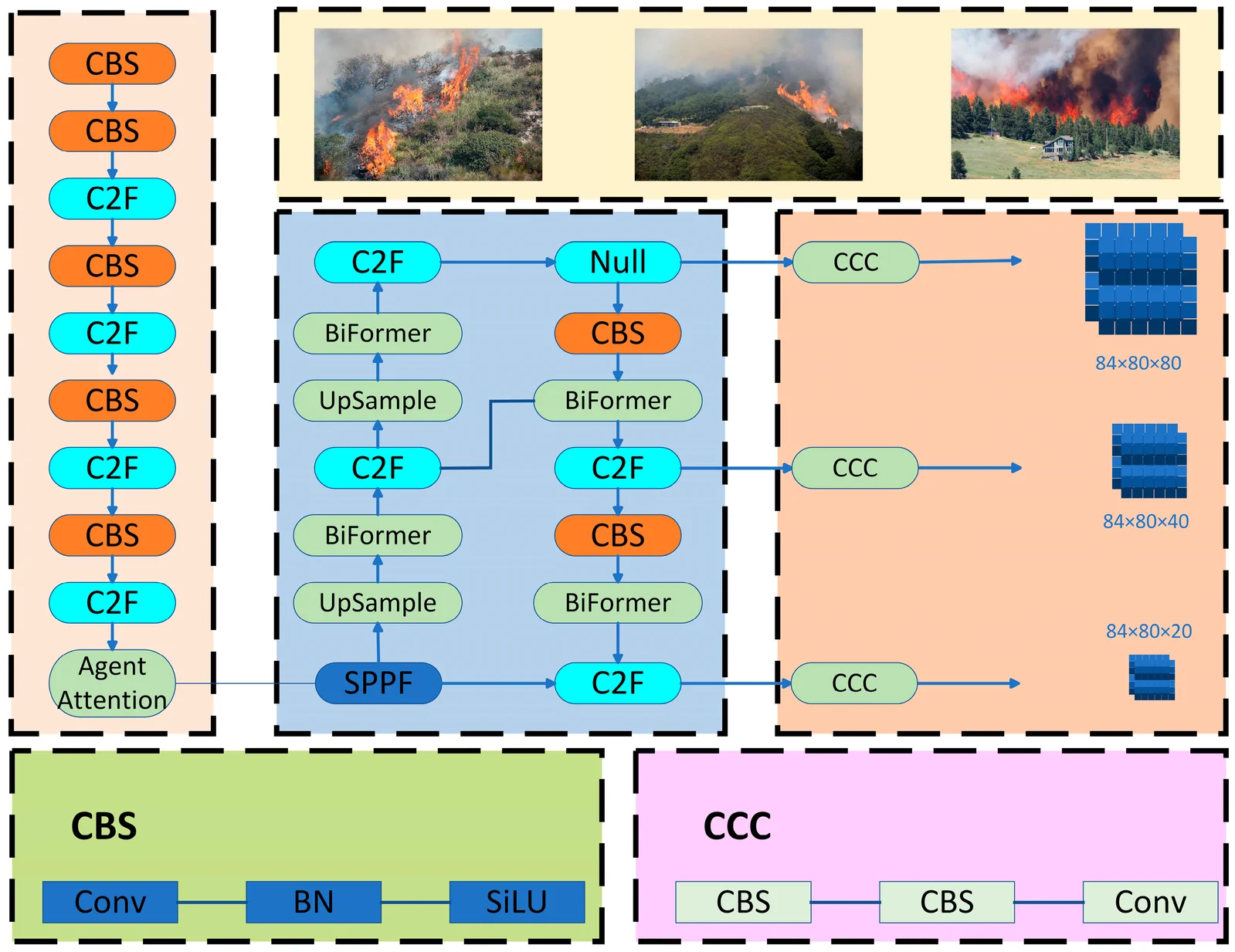
Revised Network Structure Diagram of YOLOv8.

This paper uses the modified YOLOv8 to detect the fire scene. Figure 6 shows the network architecture design principle of the improved YOLOv8n structure proposed in this paper for the fire image detection task. This architecture is based on the traditional YOLO backbone network and integrates three key improvement modules: the BiFormer Attention mechanism, the Agent Attention global perception module, and the CCC lightweight feature compression module, aiming to enhance the model’s feature extraction and accuracy performance in complex fire scenarios. The entire network structure is divided into three main parts: input encoding, feature fusion, and prediction output.

In the feature extraction stage, the input image is first processed through multiple stacked CBS modules, composed of convolution (Conv), batch normalization (BN), and activation function (SiLU), to extract low-level semantic features. At the same time, C2F modules are inserted at different layers to enhance the channel-level fine-grained information flow. The underlying structure also introduces the Agent Attention module to simulate the human visual focus mechanism, improving the model’s perception ability of the fire source area. As the network passes downward, the features enter the main part, namely the blue area in the figure, and are modeled through the BiFormer module for bidirectional feature flow, taking into account both local and global context semantics, thereby enhancing the model’s ability to distinguish heterogeneous fire targets.

In the feature fusion and upsampling stage, the multi-layer features are further stacked after upsampling and fusion, and the dimensions are compressed through lightweight C2F and CBS modules. At this time, intermediate features of different scales (80 × 80, 40 × 40, 20 × 20) enter the right CCC structure for prediction. In the CCC structure, three groups of branches composed of dual CBS modules and convolution are used to handle different-scale fire target detection tasks, respectively, effectively alleviating the target recognition errors caused by scale changes. In addition, the SPPF (Spatial Pyramid Pooling-Fast) module is used to compress semantic information and to reduce computational costs. The entire design demonstrates the characteristics of flexible structure, clear feature flow, controllable parameters, and strong deployability, and is suitable for real-time fire monitoring systems.

This paper uses four core indicators commonly used in the target detection task: Precision, Recall, AP, mAP, F1score, and FPS. They are key references for evaluating the performance of the detection model, especially suitable for the dual requirements of accuracy and completeness in safety defense scenarios such as fire detection.

(17) $$AP=\int_{0}^{1}\;P\left(R\right)dR$$

Here, AP represents the area under the Precision–Recall curve for a certain category, which is a unified metric for comprehensively evaluating Precision and Recall. During the model prediction process, by continuously adjusting the classification threshold, a series of Precision and Recall pairs can be obtained, thereby drawing the PR curve. AP integrates the curve to evaluate the model’s overall performance at different thresholds. In practical applications, the larger the AP, the better the model performs in balancing false positives and false negatives.

(18) $$mAP=\frac{1}{N}\sum_{i=1}^{N}\;AP_{i}$$

Here, mAP is the average value of AP for all detection categories, where N represents the number of categories and $AP_{i}$ represents the Average Precision of the i-th category. As the core metric for overall detection performance, mAP is widely used for model comparison. In the fire detection task, mAP can measure the overall detection level of the model for all fire-related target indications, and is a key indicator for evaluating whether the system is suitable for practical deployment.

(19) $$P=\frac{TP}{\left(TP+FP\right)}$$

Precision (P) indicates the proportion of samples predicted by the model as positive (i.e., fire sources) that are actually fire sources, TP (True Positives) refers to the cases where the prediction is a fire source and the actual situation is also a fire source, while FP (False Positives) refers to the cases where the prediction is a fire source but the actual situation is not. In a fire scenario, Precision reflects the level of false alarm rate. A high Precision means that the system rarely issues false alarms for non-fire images, which is crucial for ensuring the efficiency of emergency response. In areas with high foot traffic or high density, false alarms can cause unnecessary panic and waste of resources. Therefore, Precision must be maintained at a high level.

(20) $$R=\frac{TP}{\left(TP+FN\right)}$$

Recall (R) indicates the proportion of actual fire source images that have been successfully detected by the model and FN (False Negatives) signifies the image is actually a fire source, but the model failed to detect it. Recall reflects the situation of missed detections. In fire monitoring, missed detections are more dangerous than false alarms because a false alarm means that the system cannot promptly warn of the real fire situation, which may lead to major safety accidents. Therefore, Recall is an important metric for measuring the robustness of a fire detection system.

(21) $$F1\;score=\frac{2TP}{2TP+FP+FN}$$

The F1 Score takes into account both the False Positive (FP) and the False Negative (FN) situations of the model, and is particularly important in fire detection. Since missed detections (FN) in fire scenarios can lead to disastrous consequences, while false detections (FP) may cause unnecessary panic and waste of resources, the F1 Score provides a single metric to evaluate the model’s overall performance in these two aspects. The higher the F1 value, the better the model achieves a balance between Precision and Recall.

(22) $$FPS=\frac{1}{\mathrm{Processing}\;\mathrm{time}\;\mathrm{per}\;\mathrm{frame}}$$

In the fire monitoring system, real-time performance is of utmost importance. A high FPS (frames per second) indicates that the model can respond quickly to fire incidents and is suitable for scenarios, such as video surveillance and unmanned aerial vehicle inspection, that require high timeliness. The “Processing time per frame” represents the processing time for each frame.

## 3. Experiment

### 3.1. Image Denoising

**Figure 7:**
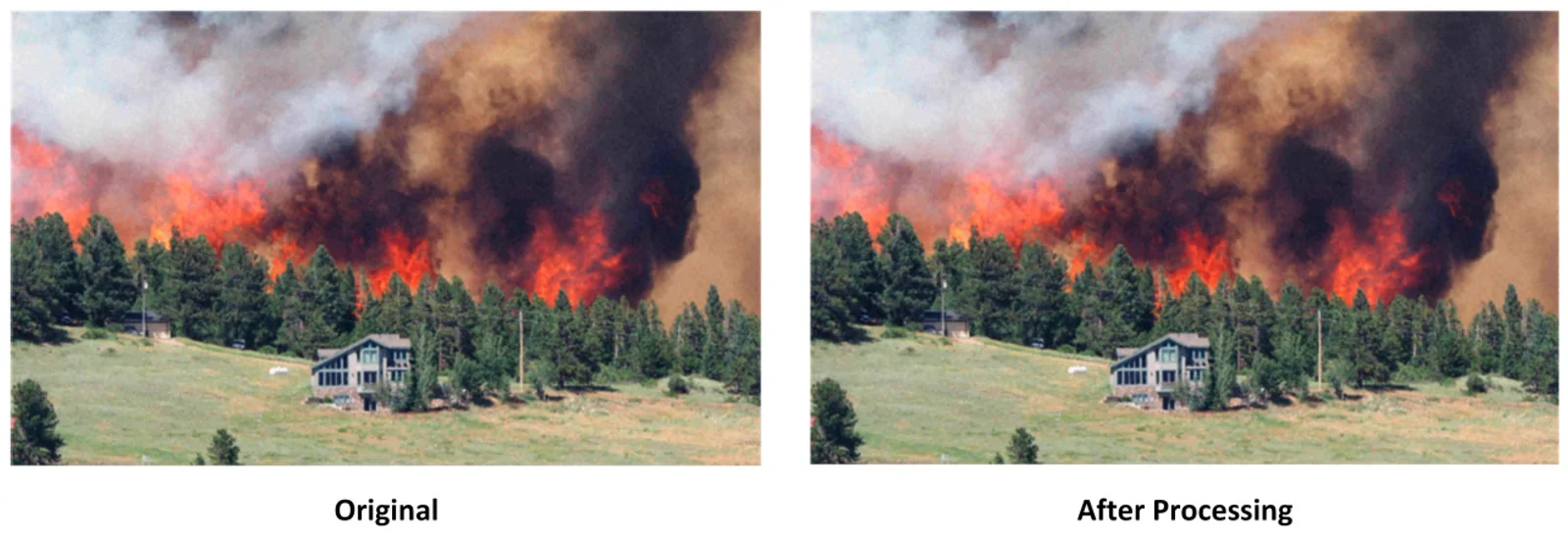
Denoising Result Image.

By analyzing the image denoising results shown in Figure 7, it is clearly observable that the image quality has been significantly improved after the processing. In the original image, due to the dense smoke and intense thermal disturbance at the fire scene, as well as the fact that the camera equipment is prone to introduce sensor noise in high-temperature and high-vibration environments, the image overall has obvious graininess, blurred edges, and missing local detail information. This noise interference not only affects the distinguishability of the flame area but interferes with the background structure (such as the edge of trees and the outline of buildings), greatly limiting the ability of the visual perception system to extract key targets.

The processed image shows higher spatial consistency and structural clarity. It can be observed that the edge contour of the flame area is sharper, the color gradation is more distinct, and the details that were previously obscured by noise, such as the forest canopy and building eaves, have been effectively restored. At the same time, the uniformity of the background has increased, the noise components have significantly decreased, and the overall visual perception of the image has become more natural, with more realistic and credible structural textures. Thus, the denoising module successfully retains the key semantic information of the fire image while effectively suppressing high-frequency disturbances, providing a more usable image input for subsequent flame detection and smoke boundary recognition.

From a system perspective, image denoising processing not only improves the quality of fire images but significantly enhances the robustness and discrimination ability of the visual model in complex environments. Especially in the early fire warning, higher-quality image input helps the model more accurately separate abnormal areas from background information, thereby reducing false alarm rates and missed detections, and improving the overall response efficiency of the system.

### 3.2. Image Blurring Modeling and Restoration

#### 3.2.1. Data Preparation

**Figure 8:**
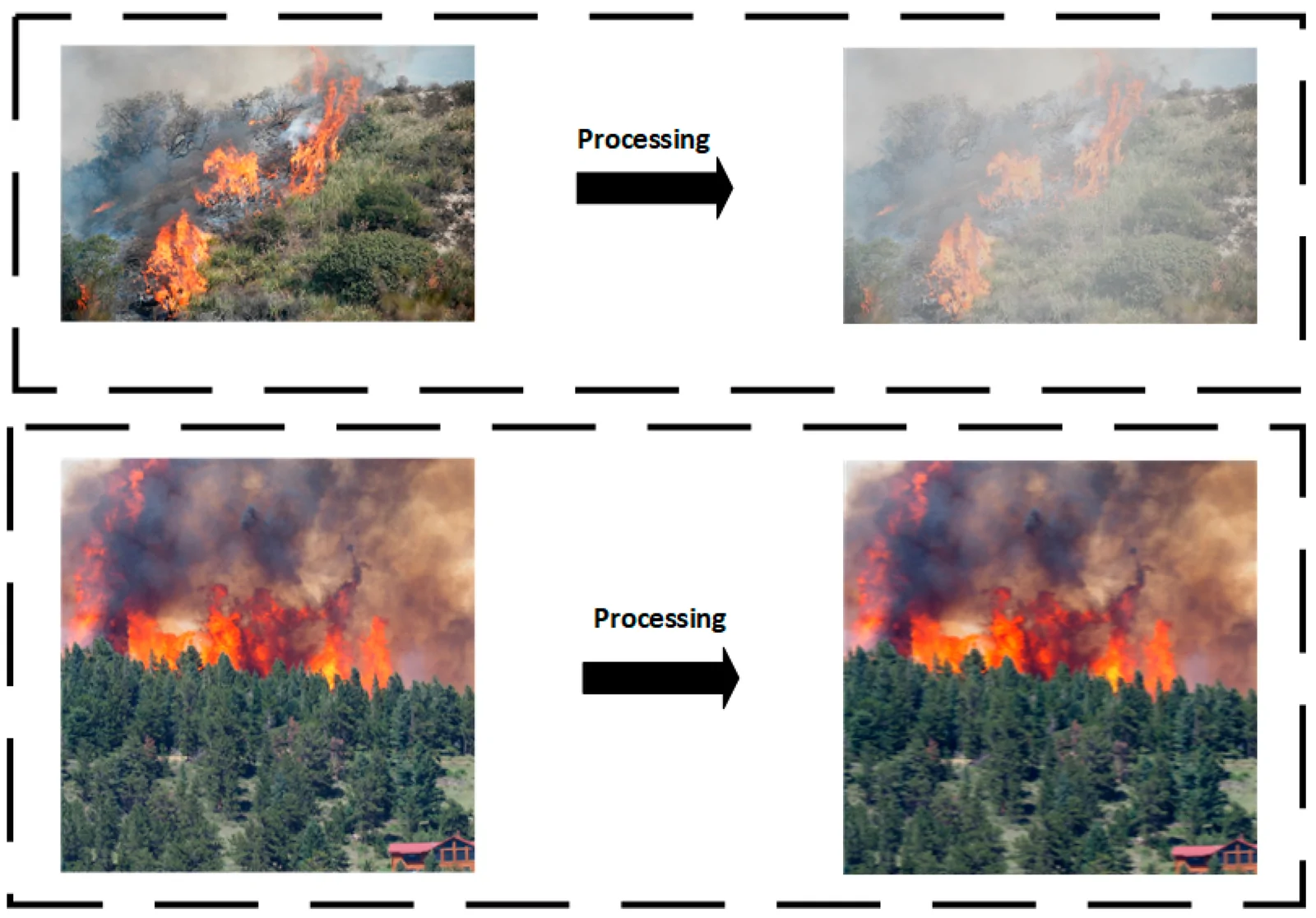
(Dynamic Blur, Fog Data) Processing Diagram.

To enhance the robustness of the visual detection model in fire scenarios under complex environments, this paper introduces targeted image degradation simulation methods during the dataset construction stage, generating image samples affected by fog interference and dynamic blurring, which truly reproduces the common visual degradation situations in fire scenes. The image on the left of Figure 8 shows the original collected image, which has good clarity, brightness, and edge information, and is an ideal basis for training samples. The image on the right of Figure 8 shows the fogged image from the same perspective. Its overall contrast has significantly decreased, and some areas have color bleaching and detail blurring phenomena, especially the information loss at the boundary between the flame and the smoke is relatively serious. Through simulating the light scattering and absorption in the atmospheric propagation process, this type of image effectively reproduces the visual blurring interference caused by smoke obstruction in the real fire scene. Further, by processing the image with the motion blur mechanism, it can be observed in the image that, due to the dynamic disturbance of the target object or the imaging device, the edges of the flame and waste materials have obvious trailing phenomena, and the texture details have been stretched or even disappeared. This spatial non-uniform blurring characteristic is extremely common in actual fire monitoring videos and poses a severe challenge to the model’s feature extraction and detection accuracy. In scenarios with rapid flame spread and rolling thick smoke, dynamic blur is prone to interfere with the target positioning module, resulting in detection errors and recognition delays.

Through the above image degradation simulation, this paper constructs training data with realistic complexity and physical credibility, significantly improving the generalization ability of the subsequent image enhancement network in the face of real complex environments. These images not only provide high-quality training data for the dehazing and deblurring models, but lay a solid foundation for the improvement of the robustness of target detection tasks, such as flames and smoke. Experiments have shown that the modified YOLOv8 detection model trained on the above degraded images has stronger environmental adaptability, especially in extreme scenarios such as insufficient lighting, smoke obstruction, or camera jitter, it can still maintain good detection performance and accuracy. The introduction of these images not only improves the usability of the overall system in real disaster scenarios, but provides important support for the construction of a multi-task collaborative fire warning visual system.

#### 3.2.2. Image Dehazing

**Figure 9:**
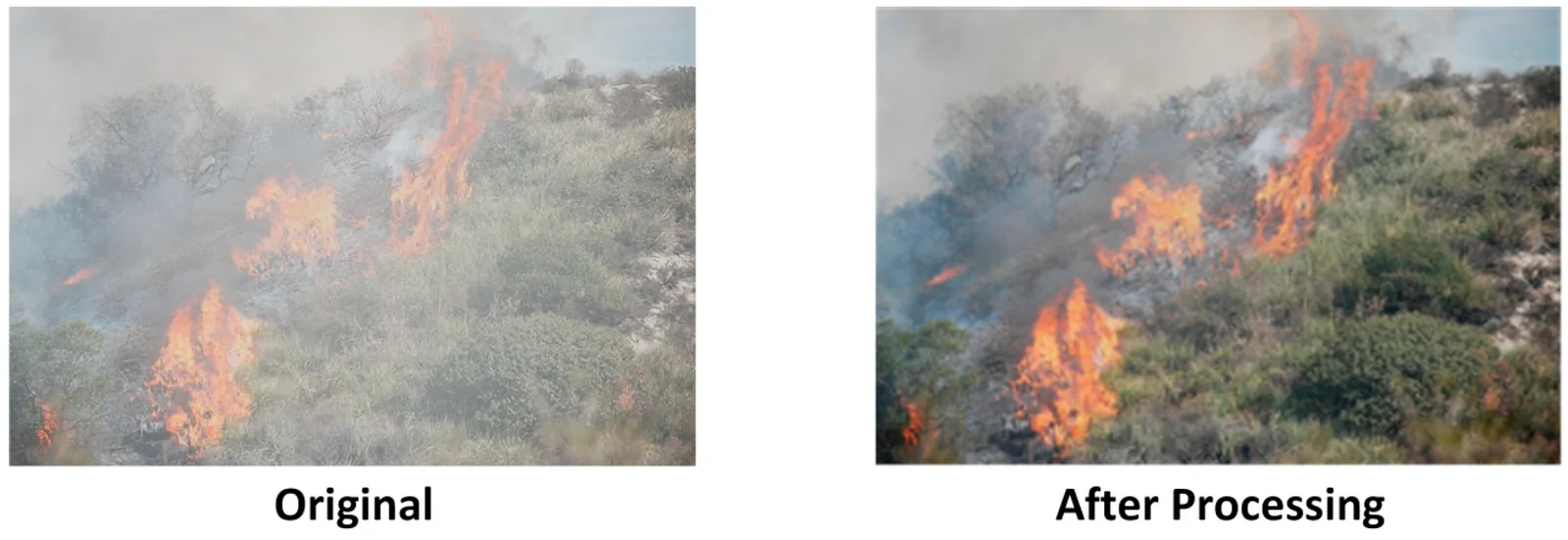
Result of Fog Removal.

In actual fire scenarios, haze has a significant impact on image quality. In severe cases, it can obscure the key features of the flame area, posing a great challenge to subsequent flame detection algorithms, as shown in Figure 9.

In the original image, the fire scene is completely covered by thick smoke, presenting a low-contrast, grayish visual effect. The boundaries of the flame area are especially blurry and the color is dim, making it difficult to separate the fire source from the background. The details of the vegetation are also blurred under the interference of the fog, and the texture information is largely lost. This degradation of image quality severely limits the precise positioning and classification ability of computer vision-based fire recognition models for the target area.

After applying the image dehazing algorithm, the clarity and gradation of the image have significantly improved. In the processed image, the edge contours of the flame area are clearer, and the orange–red flames stand out more prominently against the high-contrast background, enhancing visual saliency and facilitating the rapid locking of the fire source area by the detection model. At the same time, the details and textures of the foreground vegetation are effectively restored, with the green shrubs and soil structure clearly distinguishable. The interference of the smoke is significantly weakened, and the overall color of the image becomes more realistic, with higher visual quality.

From the perspective of image enhancement, this dehazing processing not only improves the accessibility for the identification of the target area but restores the key structure and semantic information in the image, possessing significant practical value. In the early warning system for fires, thick smoke often masks key visual features, and traditional visual detection methods are prone to missed detections or false detections. The results of this dehazing experiment show that, by using dehazing algorithms based on deep learning or physical modeling, the image degradation caused by atmospheric scattering can be effectively eliminated, improving the robustness and sensitivity of the fire detection system in complex smoke environments.

#### 3.2.3. Image Deblurring

In the tasks of fire monitoring and intelligent visual detection, the dynamic blurring issue of images is a highly challenging interference factor, especially when fire images are collected by unmanned aircraft or mobile platforms. Due to factors such as vibration, rapid movement of the target, or excessive exposure time of the equipment, spatially non-uniform blurring distortion is easily generated. To address this problem, this paper introduces and experimentally evaluates the image deblurring model based on the ChaIR architecture, conducting an in-depth analysis from the perspectives of visual quality and practical perception effects.

**Figure 10:**
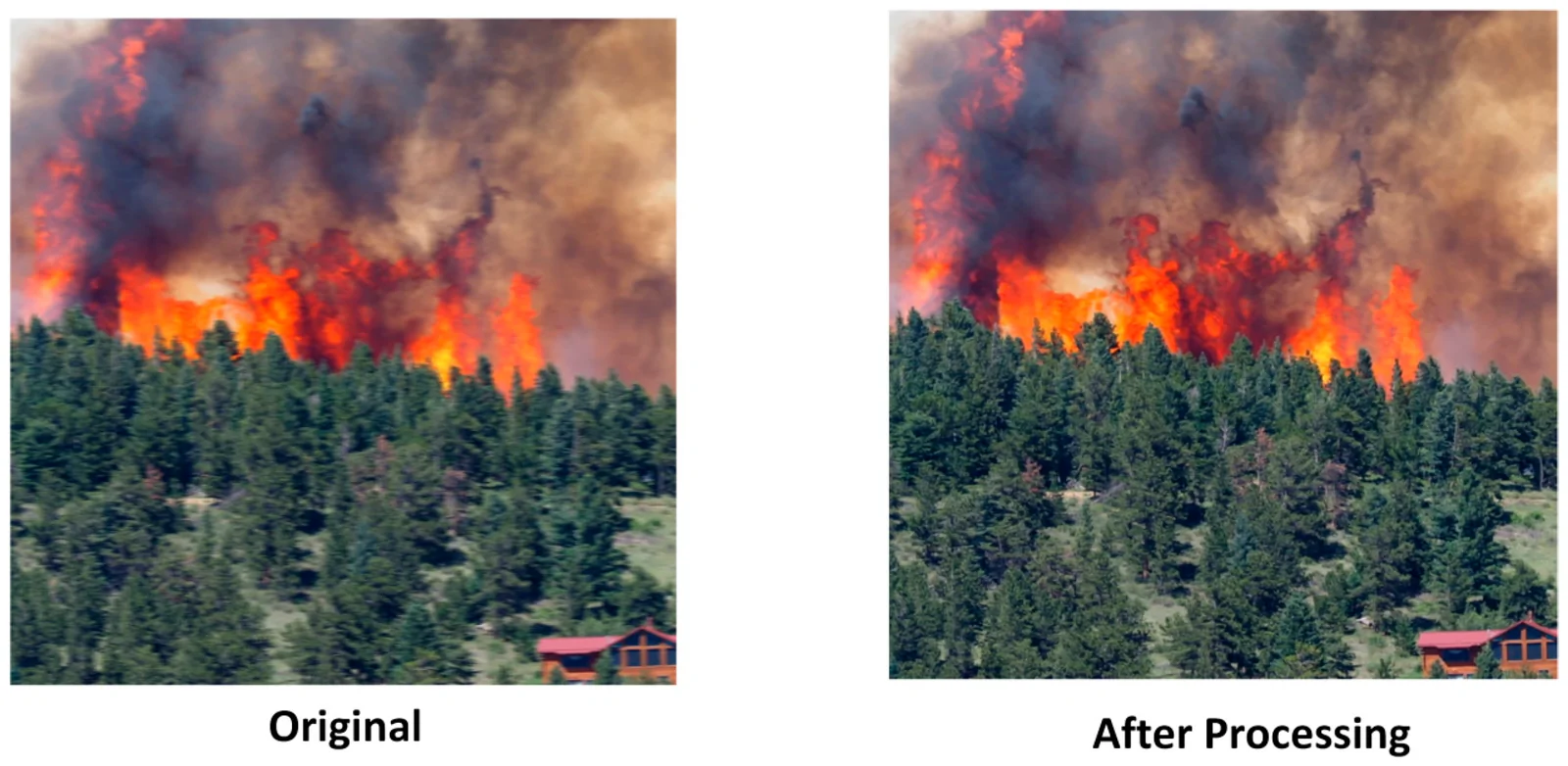
Result image after motion blurring.

As shown in Figure 10, by simulating the dynamic blurred image generated after camera shake and object movement path, it can be observed that the edges of the flame area show a significant trailing phenomenon, the boundary between the flame and the background is unclear, and the detailed garbage areas in the entire scene also exhibit structural stretching and overlapping, resulting in a significant decrease in the image’s recognition ability. On this basis, after applying the ChaIR network deblurring processing, it can be clearly seen that the artifacts caused by blurring have been largely eliminated, the edges of the flame have recovered a better clarity and continuity, and the texture information of the background area has also been structurally reconstructed. The overall image presents a visual quality approaching that of a real-shot image.

From the perspective of image content changes, compared with the previous blurred state, the restored flame area is not only more focused and sharp in shape, but has a more saturated color gradation. This means a more accurate candidate box positioning, a lower false alarm rate, and a higher recall ability for the subsequent fire detection model, significantly improving the model’s response speed and accuracy to disaster events. Moreover, ChaIR does not introduce additional artifacts or texture noise during the processing of blurred areas, maintaining the consistency of the image’s natural appearance, which meets the requirements of industrial-grade visual systems for the stability and usability of deblurring results.

### 3.3. Image Data Augmentation

This paper uses a large-scale collection of fire images generated by Projected GAN, which is trained in the NVIDIA RTX 4090 (NVIDIA Corporation, Santa Clara, California, USA) graphics card environment. In the training settings, the model configuration selects FastGAN as the basic framework. This structure ensures a fast convergence speed while having strong generative expression capabilities. The batch size is set to 64 to fully utilize the memory resources of the 4090.

The total training iterations are set to 30,000 kimg, which is 300,000 images, ensuring that the generator can fully learn at different scales and structural levels. Image augmentation adopts the x-flip strategy to expand the distribution of the training samples. Regarding the optimizers, the initial learning rates for the generator and discriminator are both set to 0.0002, and the optimizer uses Adam. To measure the generative fidelity of the model in terms of semantics and structure, the fid50k_full evaluation metric is enabled. After training, the Fréchet distance between the generated samples and the real images is calculated.

In addition, the system enables the multi-scale projection discriminators and the perceptual space alignment mechanisms, which support more stable training dynamics and higher image diversity. During the experiment, model snapshots are saved every 50 kimg, and the training status is output every 4 kimg to facilitate monitoring of the model’s convergence behavior and strategy adjustment. The overall training process achieves good convergence speed and a low FID score through this parameter combination, indicating that this network has the ability to generate real and structurally consistent samples in the fire image enhancement task. The specific parameters are shown in Table 1.

**Figure 11:**
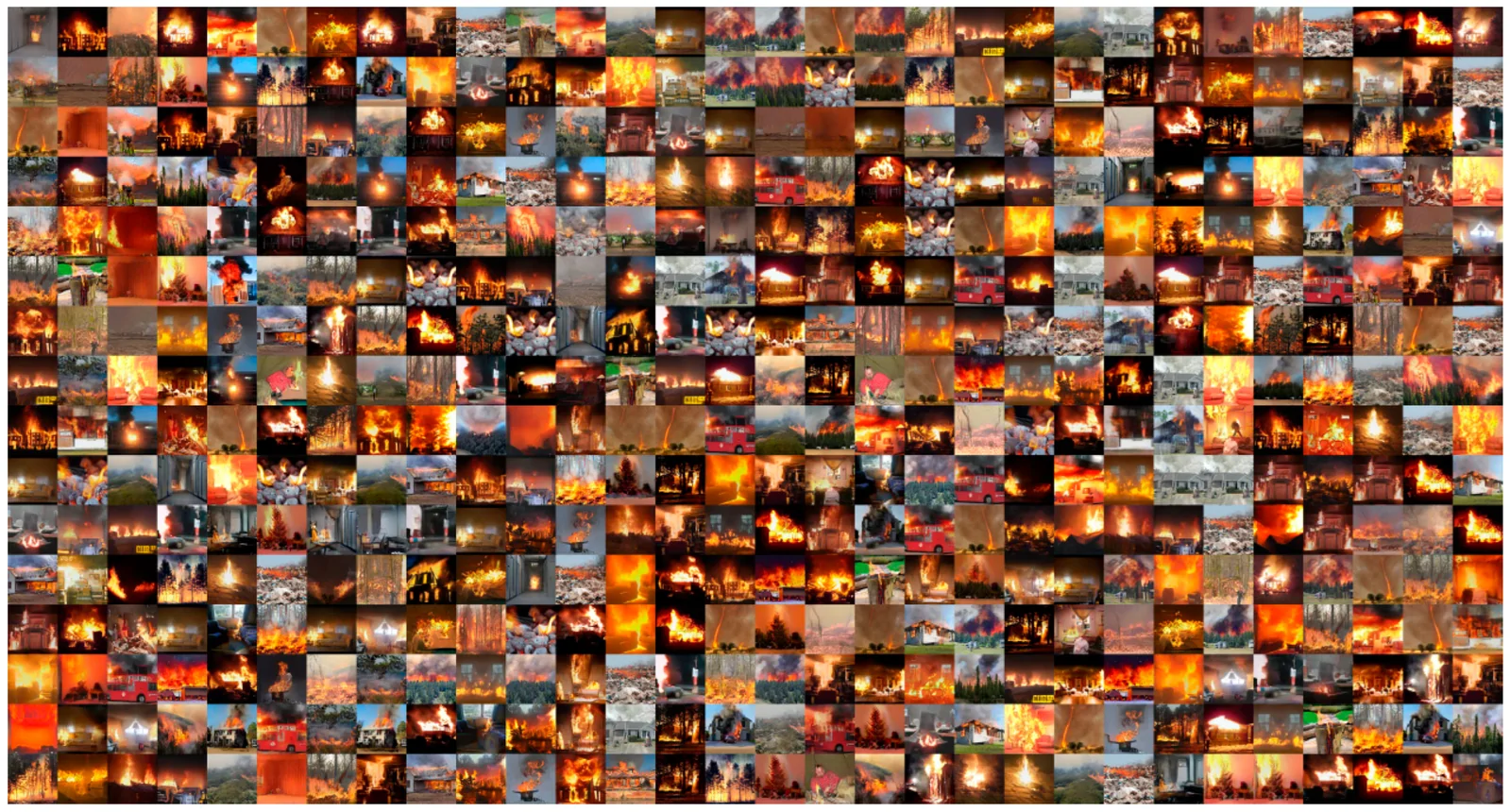
Projected GAN Generation Result Graph.

The generated image is shown in Figure 11, demonstrating the outstanding ability of this generation model in high diversity and high-quality image synthesis tasks. From an intuitive perspective, the generated images exhibit extremely high differences in aspects such as the appearance of flames, environmental scenes, brightness and tone, and composition style. They possess cross-scale, cross-scenario transferability and generalization capabilities. This ability is of great significance for constructing high-quality fire detection datasets, especially in scenarios where there are insufficient samples or data collection is limited.

From a local observation of the image, it can be found that the Projected GAN effectively captures the morphological features of the flames (such as irregular edges, dynamic flow sensation), color distribution (yellow and red overlapping, high-contrast areas centered), and typical background environments (such as buildings, mountains, night scenes, etc.) during the synthesis process, making the synthesized images visually close to the real captured images. This characteristic is attributed to its LFS structure, which linearly fuses multiple convolutional kernel bases and dynamically adjusts the fusion weights based on image content to achieve the adaptive modeling of local details. Compared to traditional StyleGAN-like methods, the LFS architecture shows particularly significant improvements in spatial consistency and semantic resolution, effectively avoiding frequent artifacts or edge confusion in the generated images.

The distribution of fire scenes in this dataset shows good balance, covering both indoor and outdoor scenarios, as well as various complex situations, such as day and night, different distances and proportions. This wide coverage of data distribution indicates that Projected GAN has strong semantic fusion and multimodal abstraction capabilities, being able to achieve diverse expressions of background, environment, and details while maintaining the consistency of core semantics (with flames as the main target). This is crucial for enhancing the robustness of the target detection network in complex environments.

### 3.4. Object Detection

The improved model of YOLOv8 was trained in the NVIDIA RTX 4090 graphics card environment. A set of optimized key training parameters was selected to achieve efficient convergence and excellent detection performance. As shown in Table 2, the input image resolution used is 640 × 640, with the number of epochs set to 200 to ensure the model has sufficient feature learning time in complex scenarios.

Considering the powerful computing resources of 4090, a batch size of 64 was used to improve training stability and to accelerate convergence speed. In terms of the optimizer, SGD (with momentum = 0.937 and weight decay = 0.0005) was adopted as the main optimization strategy, combined with an initial learning rate Lr0 = 0.01, and supplemented by warmup and cosine learning rate schedulers, which can achieve a better learning dynamic balance. Additionally, the Mosaic = 1.0 data augmentation strategy was enabled to help improve the model’s generalization ability to complex background flames, and the Scale = 0.5–1.5 setting further enhanced the model’s multi-scale adaptability.

**Figure 12:**
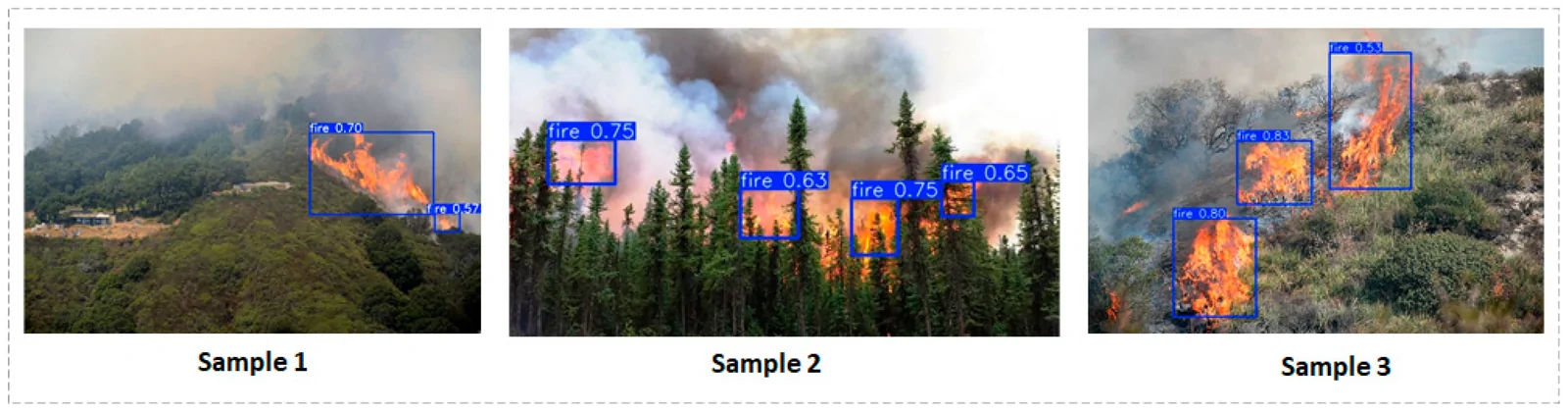
Fire Detection Result Diagram.

As shown in Figure 12, in this experiment, representative forest fire scene images were selected, covering low-vegetation areas, dense forest regions, and long-distance monitoring scenarios, aiming to evaluate the adaptability and accuracy of the fire detection model in different environments. The fire was mainly concentrated in the lower shrubbery, with clear flame edges and rich colors. The detection model was able to accurately label three areas of open flames, with confidence levels of 0.83, 0.80, and 0.53, respectively, indicating good target detection performance in this scenario, The model had a good response to high-brightness open flames, and there was less background interference and a lower false detection rate.

In the dense forest fire scene, there was a large amount of thick smoke and high-density vegetation in the background, with flames and trees interlaced. Some flame areas were obscured. Nevertheless, the detection model was still able to identify five fire points, with confidence levels ranging from 0.63 to 0.75, demonstrating the model’s robustness in complex backgrounds.

In the application scenario of fire monitoring in a long-distance observation environment, the fire points were relatively small, and the smoke was spread throughout the mountain forest area, realistically reproducing the difficulty of early detection of mountain fires. In this image, only two fire source areas were identified, with confidence levels of 0.70 and 0.57, respectively, showing a trend of a decreasing recognition rate in small target detection and in the case of long-distance blurriness. This indicates that, in such scenarios, it is necessary to combine high-resolution images, image enhancement techniques, or multi-scale feature extraction mechanisms to improve detection accuracy.

Through the qualitative analysis of the three images, the environmental robustness and diversity adaptability of the current fire detection model can be preliminarily evaluated. The excellent performance of the model under ideal conditions and its reflection of the model’s anti-interference ability in complex occlusion environments reveal that the fire monitoring application scenario in a long-distance observation environment also demonstrates strong performance under long-distance and smoke interference conditions.

## 4. Discussion

### 4.1. Image Denoising

In the study on complex scene fire detection based on machine vision, image denoising processing serves as a crucial pre-enhancement module. Fire scene images are often accompanied by intense illumination changes, thermal disturbances, smoke particle interference, and sensor noise, all of which jointly lead to a decline in image quality and further affect the accuracy and stability of subsequent tasks, such as flame edge extraction and smoke diffusion recognition. Therefore, applying high-performance denoising algorithms to pre-process the original images can significantly enhance the robustness of the visual system in extreme environments.

In the image denoising task, as shown in Table 3, KBNet performs best in both the peak signal-to-noise ratio (PSNR) and the structural similarity index (SSIM), reaching 34.67 dB and 0.968, respectively, far exceeding the other two mainstream methods, NAFNet (33.11 dB, 0.959) and PMN (33.28 dB, 0.953). An improvement in the PSNR means that the reconstructed image is closer in pixel accuracy to the original clear image, while the SSIM focuses more on the retention ability of image structure information, with a score as high as 0.968 indicating that KBNet not only removes noise but retains the image texture and edge features to the greatest extent.

In fire scenarios, this advantage is particularly crucial. High-quality images not only help improve the detection accuracy of flame shape, color, and motion features, but reduce false detection rates and missed detection rates, enhancing the perception ability of the fire warning system for abnormal heat sources and smoke diffusion boundaries. Moreover, the excellent performance of KBNet provides technical support for fire visual systems deployed on resource-constrained platforms, and its lightweight and performance-balanced characteristics are expected to be applied in real-time unmanned aerial vehicle monitoring, edge computing cameras, and other scenarios.

KBNet demonstrates significant performance advantages in the image denoising task of fire visual perception. Its ability to repair image quality in extreme environments will provide a more stable visual input foundation for intelligent fire detection systems, thereby improving the overall intelligence level and response efficiency of the security defense system.

### 4.2. Image Blurring Modeling and Restoration

In the fire detection system, the clarity and structural integrity of the image are the key prerequisites for the efficient operation of subsequent visual perception tasks. However, images in real fire environments are often disturbed by various degradation factors, such as haze coverage, smoke scattering, and camera movement, resulting in image blurring, reduced contrast, and loss of edge information. Therefore, when constructing a robust intelligent fire detection system, how to improve image quality at an early stage becomes a fundamental technical issue. This paper adopts the ChaIR network architecture based on the attention mechanism to separately handle the tasks of image dehazing and motion deblurring, and quantitatively analyzes the improvement of image quality using two standard indicators: PSNR and SSIM.

As shown in Table 4, in the research on complex scene fire detection based on computer vision, the improvement of image quality is crucial for enhancing the recognition accuracy of the system in complex environments. Fire scenes are often accompanied by a large amount of smoke and dynamic blur factors, such as smoke obscuration, thermal flow disturbance, and camera vibration. These interferences not only weaken the distinguishability of flames, smoke columns, and high-temperature areas in the image, but greatly limit the response speed and accuracy of the intelligent detection system. To address these issues, the introduction of image dehazing and motion deblurring techniques has become an important component of the fire visual enhancement process.

From the table, it can be seen that, in the dehazing task, the ChaIR algorithm performs the best, achieving a PSNR of 27.290 dB and an SSIM of 0.9792, which is much higher than DeHamer (25.913 dB, 0.9527) and FFA-Net (25.187 dB, 0.9439). This indicates that ChaIR has significant advantages in restoring the contrast, clarity, and edge structure of fire images, and can better restore key areas obscured by smoke (such as flame contours and boundaries of the trapped area), improving the reliability of fire situation detection.

In the motion deblurring task, ChaIR also leads, with a PSNR of 26.278 dB and an SSIM of 0.9613, superior to Restormer (25.181 dB, 0.9418) and MPRNet (25.981 dB, 0.9409). This result indicates that ChaIR has stronger processing ability for dynamic blur, and can effectively handle image blurring caused by camera jitter or rapid flame movement, which is conducive to improving the system’s ability to dynamically capture rapidly spreading fire situations.

In the early warning system for fires, combining dehazing and motion deblurring technologies can significantly improve image quality, thereby enhancing the perception ability of key features, such as the speed of flame spread, smoke concentration, and dynamic changes in the affected area. Especially in scenarios such as unmanned aerial vehicle inspection, remote monitoring, and edge device deployment, ChaIR, a high-performance image restoration model, provides a more stable and accurate image input foundation for the fire visual defense system, promoting the implementation of reliable and timely fire situation intelligent recognition solutions. From the trend of PSNR and SSIM, ChaIR is more suitable for processing degraded features with certain continuity and decomposability, such as scattering haze and simple blur kernels. However, when dealing with extreme motion blur, the restoration effect is still limited by the underlying feature restoration ability. Therefore, when constructing a complete fire image processing chain, it is recommended to incorporate ChaIR as a part of the image quality enhancement module, combined with data completion based on generative adversarial networks and style consistency optimization, to achieve a complete closed loop from image restoration to enhancement and detection.

In actual deployment, the ChaIR network can be embedded in the front-end image stream of the fire video monitoring system to pre-process each frame image, so that problems such as heat radiation characteristics of the flame area, light spots, and edge blurring can be effectively corrected before entering the target detection model. Especially in the YOLO high-precision flame detection model, a slight improvement in the input image quality can significantly improve recall and mAP performance. With an SSIM close to 1, ChaIR proves its excellent structural preservation performance, suitable for extreme environment perception tasks, and provides a higher-quality feature basis for subsequent detectors.

### 4.3. Image Data Augmentation

**Figure 13:**
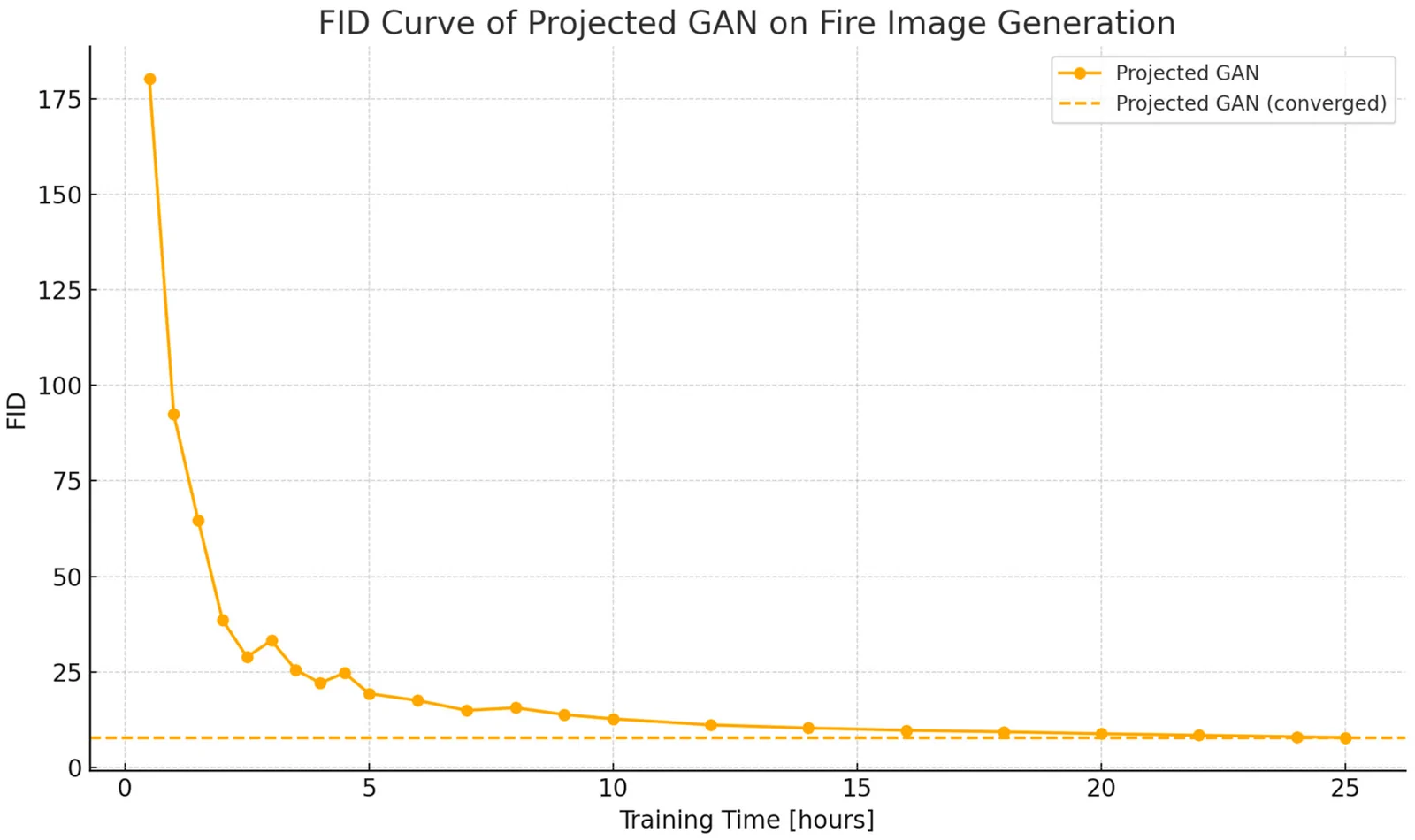
FID Evaluation Indicators.

In the experiments of this paper, we sent the generated images and the real fire images to the pre-trained Inception-v3 network, extracted the 2048-dimensional feature vectors from the second-to-last layer, and calculated the FID value according to the above formula. The experimental results are shown in Figure 13. Moreover, it demonstrated higher consistency in terms of diversity, edge integrity, and the expression of the fire target structure, verifying the superiority of this generative model in the visual synthesis scenario of fires. The FID value of Projected GAN decreased rapidly in the early training stage, and reached a stable convergence after approximately 20 h, being lower than 10.

This trend indicates that this model has a strong ability to learn complex fire-related features. Integrating such high-quality synthetic fire images into the training process of the fire detection network can enhance its robustness and generalization ability, especially in conditions involving smoke, blurriness, and occlusion. Therefore, in the field of fire detection based on computer vision, the projection-based generative adversarial network has made significant contributions to the construction of a reliable intelligent fire alarm system.

### 4.4. Object Detection

**Figure 14:**
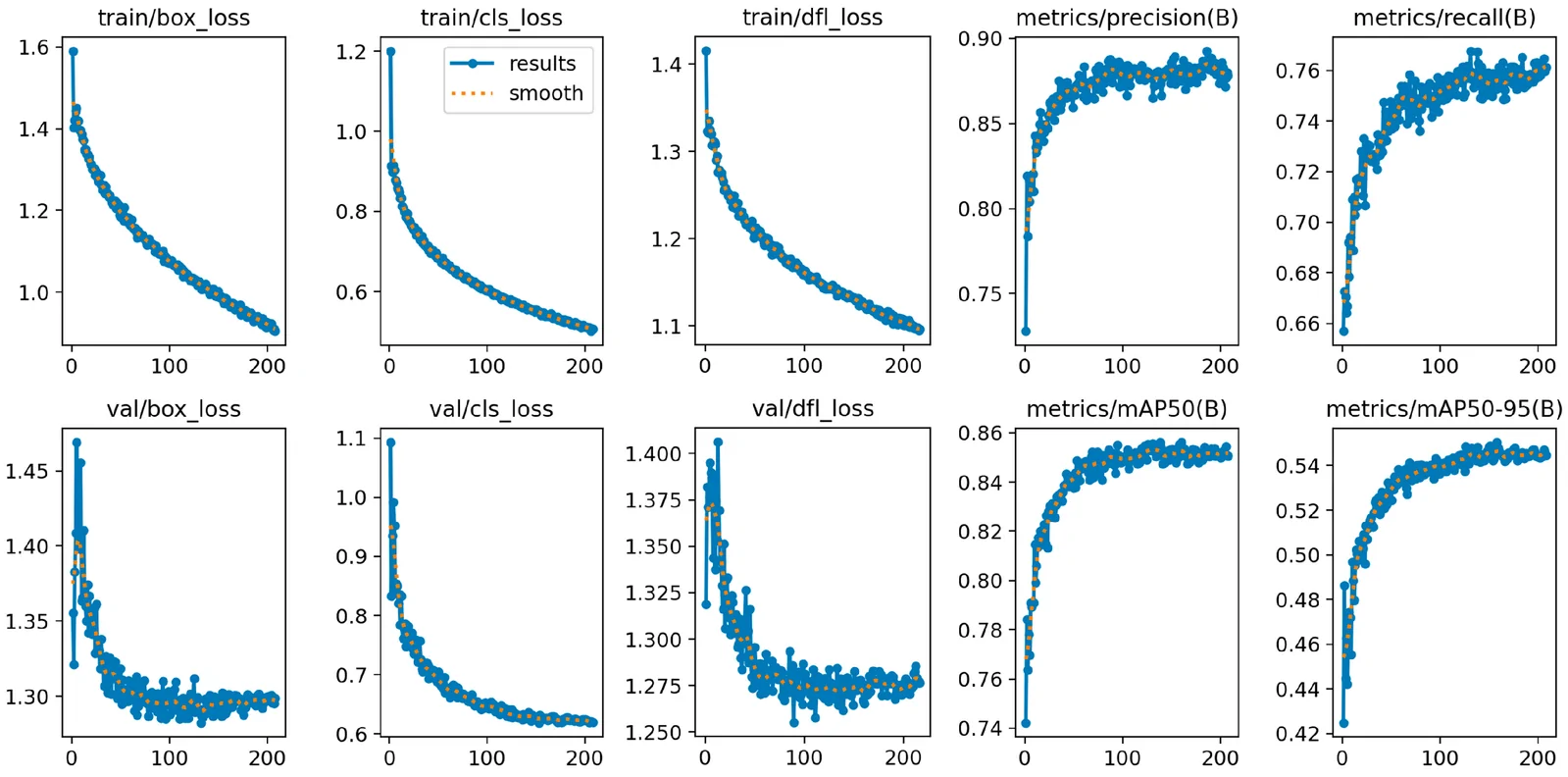
Training Process Diagram of Revised YOLOv8.

The training process of YOLOv8n and its enhanced models in the fire image detection task is shown in Figure 14. It demonstrates excellent convergence. During the entire 200 epoch training process, various loss functions, such as train/box_loss, train/cls_loss, and train/dfl_loss, all show a monotonically decreasing trend. The initial stage of training has a faster decline rate, and then it tends to stabilize. This reflects that the network structure gradually stabilizes in terms of target positioning, classification discrimination, and distance modeling. On the validation set, val/box_loss and val/cls_loss also show a continuous decline with a relatively small fluctuation range, indicating that the model has good generalization ability while avoiding overfitting. In addition, the overall trend of indicators related to detection performance, such as metrics/precision (B), metrics/recall (B), and metrics/mAP50 (B), shows a significant upward trend, and converges to a higher level in the later stage, indicating that the designed model has strong target recognition ability and stability in fire images.

The experimental results of model performance comparison under different module combinations are shown in Table 5. It can be seen that the improved scheme proposed in this paper, after introducing the CCC, BiFormer structure, and Agent Attention module, has shown significant advantages in the core indicators of the target detection task. This verifies the effectiveness of multi-module fusion in improving detection accuracy and robustness.

In terms of Precision, the final model integrating all modules (our model) reaches 0.891, which is significantly higher than the basic YOLOv8 model (0.866) and also higher than the BiFormer + Agent Attention combination without introducing the CCC (0.875). This result indicates that the CCC plays a key role in further compressing information redundancy and strengthening the perception of foreground targets, enabling the model to have stronger discrimination ability in scenarios with high background interference, such as fires, significantly reducing the false detection rate. In terms of Recall performance, our model also reaches the optimal value of 0.764, which is higher than the model containing all modules but without the CCC (Recall is 0.749), and also significantly better than the BiFormer model without Agent Attention (0.730). This improvement indicates that the CCC and the attention mechanism have a synergistic enhancing effect in modeling key spatial regions, effectively alleviating the missed detection problem and enhancing the response ability to small fire points and blurred edge targets. In terms of mAP indicators, our model reaches 0.858, ranking first among all comparison models, and is nearly 1.9% higher than the closest suboptimal combination, further verifying the end-to-end performance improvement brought about by module fusion. Additionally, the parameter size (30.42 × 10^5^) is within a medium range, and the inference speed still falls within the category of lightweight models, ensuring the practicality of the model in edge deployment scenarios.

Based on the performance comparison results of different target detection models shown in Table 6, it can be seen that the improved model proposed in this paper demonstrates strong comprehensive performance in the four key evaluation indicators of Precision, Recall, mAP, and F1, outperforming the current mainstream lightweight detection models, such as YOLOv5l, YOLOv6m, YOLOv10l, and YOLOv11l. It ensures detection accuracy while balancing the inference speed and parameter size, demonstrating high practicality and robustness.

In terms of Precision, our model reaches 0.891, slightly lower than YOLOv5l (0.897) and YOLOv10l (0.913), but still within the high-precision range, indicating that it has a lower false alarm rate in actual detection and can effectively handle non-fire interference targets in complex backgrounds, ensuring the credibility of the identification results. In terms of Recall performance, our model achieves 0.764, superior to YOLOv5l (0.662) and YOLOv10l (0.633), slightly lower than YOLOv6m (0.781) and YOLOv11l (0.771), but still within the high-recall range, showing good robustness in target coverage ability, and being able to capture fire or abnormal areas as much as possible, reducing the risk of missed detection. In terms of mAP, our model reaches 0.858, significantly higher than YOLOv5l (0.839) and YOLOv10l (0.812), slightly higher than YOLOv6m (0.851), and close to YOLOv11l (0.888), indicating its competitive overall detection accuracy in multi-scale and multi-class target detection tasks. While maintaining a superior mAP, our model significantly outperforms all comparison models in inference speed (FPS = 65.85), compared to YOLOv11l (53.70) and YOLOv6m (49.45), with an improvement rate of 22.7% and 33.1%, respectively, demonstrating stronger real-time performance and deployment efficiency. In terms of the F1 metric, our model achieves 0.822, ranking first among all models, with advantages over YOLOv6m (0.804) and YOLOv11l (0.797), further indicating that it has achieved a better balance between detection accuracy and comprehensiveness, enhancing the model’s overall adaptability in high-risk scenarios such as real fire detection.

To further verify the superiority of the proposed model, it was compared with the current mainstream fire target detection models under the same experimental conditions. The specific performance comparison results are shown in Table 7.

In terms of accuracy (Precision), our model achieved a score of 0.891, ranking first among all models. It significantly outperformed the traditional lightweight detection models SSD (0.858) and EdgeFireSmoke++ (0.856), as well as the more powerful Fire-Net (0.884) and Faster R-CNN (0.866). This result indicates that the improved model has higher robustness in distinguishing fire targets from background noise (such as smoke, spots, and structural edges), effectively reducing false alarms. In terms of Recall, our model reached 0.764, which was approximately 7 percentage points higher than that of SSD and EdgeFireSmoke++ (0.695), indicating its stronger ability to capture small, occluded, or blurred fire targets, reducing missed detections, and meeting the high requirements of actual fire warning systems for coverage. In terms of the comprehensive detection accuracy metric (mAP), our model achieved 0.858, significantly higher than Faster R-CNN (0.782) and Fire-Net (0.834), and surpassing the traditional methods SSD and EdgeFireSmoke++ (both 0.808). This indicates that the model has stronger positioning ability for different scales and shapes of fire sources, suitable for complex and unstructured fire environments. The F1 metric, as a balance metric of Precision and Recall, achieved 0.822, significantly higher than Fire-Net (0.798) and Faster R-CNN (0.796), indicating a better balance between detection accuracy and completeness, and being able to achieve a better trade-off between warning reliability and comprehensive response in actual deployment. In terms of inference speed, our model reached 65.85 FPS, far exceeding EdgeFireSmoke++ (51.8), SSD (46.8), and the traditional two-stage detection framework Faster R-CNN (12.1 FPS). At the same time, the parameter size of our model was controlled at 30.42 × 10^5^, much smaller than Faster R-CNN (413.8 × 10^5^), SSD (241.2 × 10^5^), and Fire-Net (85.2 × 10^5^), indicating that it significantly reduces computational overhead while ensuring performance, greatly enhancing the feasibility of edge deployment and real-time response.

The model test results on multiple different datasets are shown in Table 8. The improved model proposed in this paper demonstrates superior performance in terms of consistency and stability across datasets, verifying its good generalization ability and robustness in multi-source and complex fire scenarios. Regardless of whether it is on the local collected dataset or the public standard fire detection set, our model achieves leading key performance indicators. On the Owns dataset, the Precision of our model reaches 0.891, Recall is 0.764, and mAP is 0.858, maintaining the best among all comparison models. Here, Precision is significantly higher than YOLOv6m (0.871) and YOLOv11l (0.837), while Recall is higher than YOLOv5l (0.662) and YOLOv10l (0.633). On the forest-fire-detection-gkpap dataset, our model also achieved the highest Recall (0.798) and mAP (0.841), with a Precision of 0.768, remaining at an excellent level. Compared with YOLOv10l (mAP = 0.837) and YOLOv11l (mAP = 0.832), although the accuracy is similar, our model has a significant improvement in Recall, indicating that the model can effectively capture more potential fire points in more challenging scenarios, such as forest fires, significantly reducing the risk of missed detections, and is suitable for actual forest fire monitoring and response scenarios.

On the CVPR Lab Fire+COCO dataset, our model achieved excellent results, with a Precision of 0.772, Recall of 0.726, and mAP of 0.769. In the Recall metric especially, it leads YOLOv5l (0.685) and YOLOv6m (0.70), and mAP exceeds YOLOv10l (0.763) and YOLOv11l (0.76). This fully demonstrates that the model still maintains good detection performance in the public mixed data scenarios, with stronger cross-domain generalization ability and model stability.

After comparing multiple variants of the YOLO series, in cases where some models have slightly higher accuracy but insufficient recall rate, or have acceptable recall ability but more false detections, this model achieves a good balance between accuracy and comprehensiveness.

## 5. Conclusions

This paper presents a multi-module fire detection framework integrating image enhancement and improved YOLOv8 object detection, aiming to address the issues of insufficient detection accuracy and poor robustness in fire early warning systems under complex visual environments. The main conclusions drawn in this paper are as follows:

1. The framework proposed in this paper significantly outperforms traditional methods in terms of PSNR (34.67 dB) and SSIM (0.968) image enhancement metrics, and achieves excellent performance in fire target detection compared to mainstream models, with the average detection accuracy (mAP) reaching up to 0.858.
2. Compared with existing fire detection methods based on the YOLO series and mainstream models, the improved YOLO proposed in this paper significantly improves the detection accuracy in complex environments while maintaining the detection speed. Based on the evaluation of the forest-fire-detection-qkpap_dataset and the CVPR Lab Fire+COCO public datasets, in the presence of transparent smoke interference and low brightness scenarios, it has improved by approximately 7.2% compared to the standard YOLOv8, and is comprehensively superior to mainstream fire detection models.
3. This system is efficient, modular, and lightweight, and is suitable for deployment in scenarios with high requirements for response speed and resource limitations, such as urban surveillance, forest inspection, and unmanned aerial vehicle platforms. It has good engineering feasibility and scalability.

Future improvements will be made in the following aspects: 1. Integrating infrared thermal imaging and multi-modal perception technologies to enhance the system’s environmental adaptability; 2. The current version has not undergone cross-scenario verification or long-term operation tests. Therefore, in subsequent research, we will introduce more datasets, conduct scene adaptability experiments, and improve the system’s stability and generalization ability in dynamic environments; 3. Combining edge computing and model compression strategies to enable real-time deployment of the system on embedded platforms.

## Figures and Tables

**Table 1:** Projected GAN Configuration Parameters.

Parameter	Vale
cfg	FastGAN
batch	64
kimg	30,000
snap	50
tick	4
glr	0.0002
dlr	0.0002

**Table 2:** Configuration of YOLO Training Parameters.

Parameter	Value
Image size	640 × 640
Epochs	200
Batch size	64
Lr0	0.01
Optimizer	SGD
Mosaic	1.0
Scale	0.5–1.5

**Table 3:** Evaluation Indicators for Denoising Results.

Task	Algorithm	PSNR (dB) ↑	SSIM ↑
Denoise	KBNet	34.67	0.968
	NAFNet	33.11	0.959
	PMN	33.28	0.953

**Table 4:** Evaluation Indicators for Denoising/Dynamic Blur Removal Results.

Task	Algorithm	PSNR (dB) ↑	SSIM ↑
Dehaze	ChaIR	27.290	0.9792
	DeHamer	25.913	0.9527
	FFA-Net	25.187	0.9439
Motion Deblurring	ChaIR	26.278	0.9613
	Restormer	25.181	0.9418
	MPRNet	25.981	0.9409

**Table 5:** Comparison of ablation experiments for each module.

YOLOv8n	CCC	BiFormer	Agent Attention	Precision	Recall	mAP	Params × 10^5^	FPS
√				0.866	0.735	0.782	37.89	56.32
√	√			0.875	0.749	0.801	36.53	60.43
√		√		0.868	0.721	0.827	35.64	60.44
√			√	0.879	0.717	0.830	34.52	62.61
√	√	√		0.884	0.729	0.834	33.88	63.69
√	√		√	0.871	0.736	0.838	32.55	62.13
√		√	√	0.869	0.732	0.833	31.71	63.71
√	√	√	√	0.891	0.764	0.858	30.42	65.85

**Table 6:** Comparison of results of different YOLO models.

Models	Precision	Recall	mAP	F1	FPS	Params × 10^5^
YOLOv5l	0.897	0.662	0.839	0.778	42.37	21.2
YOLOv6m	0.871	0.781	0.851	0.804	49.45	35
YOLOv10l	0.913	0.633	0.812	0.763	49.43	29.5
YOLOv11l	0.837	0.771	0.888	0.797	53.70	25.3
Ours	0.891	0.764	0.858	0.822	65.85	30.42

**Table 7:** Comparison with other mainstream object detection models.

Models	Precision	Recall	mAP	F1	FPS	Params × 10^5^
SSD	0.858	0.695	0.808	0.767	46.8	241.2
Faster R-CNN	0.866	0.735	0.782	0.796	12.1	413.8
EdgeFireSmoke++	0.856	0.695	0.808	0.768	51.8	33.5
Fire-Net	0.884	0.729	0.834	0.798	40.1	85.2
Ours	0.891	0.764	0.858	0.822	65.85	30.42

**Table 8:** Improvement of the performance of the YOLOv8 model on different datasets.

Dateset	Owns			Forest-Fire-Detection-qkpap_Dataset			CVPR Lab Fire+COCO		
Models	Precision	Recall	mAP	Precision	Recall	mAP	Precision	Recall	mAP
YOLOv5l	0.897	0.662	0.839	0.743	0.627	0.796	0.807	0.693	0.785
YOLOv6m	0.871	0.781	0.851	0.794	0.688	0.823	0.772	0.701	0.803
YOLOv10l	0.913	0.633	0.812	0.757	0.758	0.784	0.833	0.713	0.821
YOLOv11l	0.837	0.771	0.888	0.822	0.748	0.811	0.853	0.716	0.832
Ours	0.891	0.764	0.858	0.768	0.738	0.791	0.772	0.721	0.769

## Data Availability

The original contributions presented in this study are included in the article. Further inquiries can be directed to the corresponding author.

## References

[1] Chagger R., Smith D. (2014). The causes of false fire alarms in buildings. Brief. Pap..

[2] Podrzaj P., Hashimoto H. (2006). Intelligent space as a fire detection system. Proceedings of the 2006 IEEE International Conference on Systems, Man and Cybernetics.

[3] Chen T.H., Wu P.H., Chiou Y.C. (2004). An early fire-detection method based on image processing. Proceedings of the 2004 International Conference on Image Processing, 2004. ICIP’04.

[4] Dimitropoulos K., Gunay O., Kose K., Erden F., Chaabene F., Tsalakanidou F., Grammalidis N., Cetin E. (2012). Flame detection for video-based early fire warning for the protection of cultural heritage. Progress in Cultural Heritage Preservation: 4th International Conference, EuroMed 2012, Limassol, Cyprus, 29 October–3 November 2012.

[5] Celik T., Demirel H. (2009). Fire detection in video sequences using a generic color model. Fire Saf. J..

[6] Celik T. (2010). Fast and efficient method for fire detection using image processing. ETRI J..

[7] Marbach G., Loepfe M., Brupbacher T. (2006). An image processing technique for fire detection in video images. Fire Saf. J..

[8] Kim D.K., Wang Y.F. (2009). Smoke detection in video. Proceedings of the 2009 WRI World Congress on Computer Science and Information Engineering.

[9] Yamagishi H., Yamaguchi J. (1999). Fire flame detection algorithm using a color camera. MHS’99, Proceedings of the 1999 International Symposium on Micromechatronics and Human Science (Cat. No. 99TH8478), Nagoya, Japan, 23–26 November 1999.

[10] Lv Z., Wang F., Cui G., Benediktsson J.A., Lei T., Sun W. (2022). Spatial–spectral attention network guided with change magnitude image for land cover change detection using remote sensing images. IEEE Trans. Geosci. Remote Sens..

[11] Chen J., Li R., Tao M., Wang L., Lin C., Wang J., Wang L., Wang Y., Chen L. (2022). Overview of the performance of satellite fire products in China: Uncertainties and challenges. Atmos. Environ..

[12] Thangavel K., Spiller D., Sabatini R., Marzocca P., Esposito M. (2022). Near real-time wildfire management using distributed satellite system. IEEE Geosci. Remote Sens. Lett..

[13] Dowell D.C., Alexander C.R., James E.P., Weygandt S.S., Benjamin S.G., Manikin G.S., Blake B.T., Brown J.M., Olson J.B., Hu M. (2022). The High-Resolution Rapid Refresh (HRRR): An hourly updating convection-allowing forecast model. Part I: Motivation and system description. Weather. Forecast..

[14] Abdusalomov A., Umirzakova S., Bakhtiyor Shukhratovich M., Mukhiddinov M., Kakhorov A., Buriboev A., Jeon H.S. (2024). Drone-Based Wildfire Detection with Multi-Sensor Integration. Remote Sens..

[15] Sharma J., Granmo O.C., Goodwin M., Fidje J.T. (2017). Deep convolutional neural networks for fire detection in images. Engineering Applications of Neural Networks: 18th International Conference, EANN 2017, Athens, Greece, 25–27 August 2017.

[16] Muhammad K., Ahmad J., Mehmood I., Rho S., Baik S.W. (2018). Convolutional neural networks based fire detection in surveillance videos. IEEE Access.

[17] Zhang Q., Xu J., Xu L., Guo H. (2016). Deep convolutional neural networks for forest fire detection. Proceedings of the 2016 International Forum on Management, Education and Information Technology Application.

[18] Hassan E., El-Rashidy N. (2022). Mask R-CNN models. Nile J. Commun. Comput. Sci..

[19] Girshick R. Fast r-cnn. Proceedings of the IEEE International Conference on Computer Vision.

[20] Redmon J., Divvala S., Girshick R., Farhadi A. You only look once: Unified, real-time object detection. Proceedings of the IEEE Conference on Computer Vision and Pattern Recognition.

[21] Liu W., Anguelov D., Erhan D., Szegedy C., Reed S., Fu C.Y., Berg A.C. (2016). Ssd: Single shot multibox detector. Computer Vision–ECCV 2016: 14th European Conference, Amsterdam, The Netherlands, 11–14 October 2016.

[22] Wu S., Zhang L. (2018). Using popular object detection methods for real time forest fire detection. Proceedings of the 2018 11th International Symposium on Computational Intelligence and Design (ISCID).

[23] Seydi S.T., Saeidi V., Kalantar B., Ueda N., Halin A.A. (2022). Fire-Net: A Deep Learning Framework for Active Forest Fire Detection. J. Sens..

[24] Almeida J.S., Jagatheesaperumal S.K., Nogueira F.G., de Albuquerque V.H.C. (2023). EdgeFireSmoke++: A novel lightweight algorithm for real-time forest fire detection and visualization using internet of things-human machine interface. Expert Syst. Appl..

[25] Zhao L., Zhi L., Zhao C., Zheng W. (2022). Fire-YOLO: A small target object detection method for fire inspection. Sustainability.

[26] Khan Z.A., Ullah F.U.M., Yar H., Ullah W., Khan N., Kim M.J., Baik S.W. (2025). Optimized cross-module attention network and medium-scale dataset for effective fire detection. Pattern Recognit..

[27] Wang H., Zhang Y., Zhu C. (2025). YOLO-LFD: A Lightweight and Fast Model for Forest Fire Detection. Comput. Mater. Contin..

[28] Li X., Yan G.P., Li X.M., Chen L. (2007). Image denoise based on soft-threshold and edge enhancement. Proceedings of the Second Workshop on Digital Media and Its Application in Museum & Heritages (DMAMH 2007).

[29] Chen F.Q., Zhou Y.P. (2015). Color feature extraction of Hainan Li brocade image based on RGB and HSV. Proceedings of the 2015 12th International Computer Conference on Wavelet Active Media Technology and Information Processing (ICCWAMTIP).

[30] Patanavijit V. (2015). The bilateral denoising performance influence of window, spatial and radiometric variance. Proceedings of the 2015 2nd International Conference on Advanced Informatics: Concepts, Theory and Applications (ICAICTA).

[31] Thapa D., Raahemifar K., Lakshminarayanan V. (2015). Reduction of speckle noise from optical coherence tomography images using multi-frame weighted nuclear norm minimization method. J. Mod. Opt..

[32] Majumdar A. (2018). Blind denoising autoencoder. IEEE Trans. Neural Netw. Learn. Syst..

[33] Devi T.G., Patil N. (2020). Analysis & evaluation of Image filtering Noise reduction technique for Microscopic Images. Proceedings of the 2020 International Conference on Innovative Trends in Information Technology (ICITIIT).

[34] Yuan F., Xiao F., Zhang K., Huang Y., Cheng E. (2021). Noise reduction for sonar images by statistical analysis and fields of experts. J. Vis. Commun. Image Represent..

[35] Sun Z., Zhang Y., Bao F., Wang P., Yao X., Zhang C. (2022). Sadnet: Semi-supervised single image dehazing method based on an attention mechanism. ACM Trans. Multimed. Comput. Commun. Appl. (TOMM).

[36] Liang S., Gao T., Chen T., Cheng P. (2024). A remote sensing image dehazing method based on heterogeneous priors. IEEE Trans. Geosci. Remote Sens..

[37] Liao M., Lu Y., Li X., Di S., Liang W., Chang V. (2024). An unsupervised image dehazing method using patch-line and fuzzy clustering-line priors. IEEE Trans. Fuzzy Syst..

[38] Guo X., Yang Y., Wang C., Ma J. (2022). Image dehazing via enhancement, restoration, and fusion: A survey. Inf. Fusion.

[39] Liu J., Wang S., Wang X., Ju M., Zhang D. (2021). A review of remote sensing image dehazing. Sensors.

[40] Yang G., Evans A.N. (2021). Improved single image dehazing methods for resource-constrained platforms. J. Real-Time Image Process..

[41] Xu L., Wei Y. (2022). “Pyramid deep dehazing”: An unsupervised single image dehazing method using deep image prior. Opt. Laser Technol..

[42] Li R., Cao H., Fan Y., Cai C., Zhang S., Xue H., Zeng Q. (2024). Multi-Indicator reconstruction for underwater polarized image dehazing method. Opt. Lasers Eng..

[43] Tsai F.J., Peng Y.T., Lin Y.Y., Tsai C.C., Lin C.W. (2022). Stripformer: Strip transformer for fast image deblurring. Proceedings of the European Conference on Computer Vision.

[44] Kong L., Dong J., Ge J., Li M., Pan J. Efficient frequency domain-based transformers for high-quality image deblurring. Proceedings of the IEEE/CVF Conference on Computer Vision and Pattern Recognition.

[45] Dong J., Pan J., Yang Z., Tang J. Multi-scale residual low-pass filter network for image deblurring. Proceedings of the IEEE/CVF International Conference on Computer Vision.

[46] Ren M., Delbracio M., Talebi H., Gerig G., Milanfar P. Multiscale structure guided diffusion for image deblurring. Proceedings of the IEEE/CVF International Conference on Computer Vision.

[47] Ji S.W., Lee J., Kim S.W., Hong J.P., Baek S.J., Jung S.W., Ko S.J. XYDeblur: Divide and conquer for single image deblurring. Proceedings of the IEEE/CVF Conference on Computer Vision and Pattern Recognition.

[48] Kim K., Lee S., Cho S. (2022). Mssnet: Multi-scale-stage network for single image deblurring. Proceedings of the European Conference on Computer Vision.

[49] Matlin E., Milanfar P. Removal of haze and noise from a single image. Computational Imaging X.

[50] Fang F., Li F., Zeng T. (2014). Single image dehazing and denoising: A fast variational approach. SIAM J. Imaging Sci..

[51] Shin Y.S., Cho Y., Pandey G., Kim A. (2016). Estimation of ambient light and transmission map with common convolutional architecture. Proceedings of the OCEANS 2016 MTS/IEEE Monterey.

[52] Perez J., Attanasio A.C., Nechyporenko N., Sanz P.J. (2017). A deep learning approach for underwater image enhancement. Biomedical Applications Based on Natural and Artificial Computing: International Work-Conference on the Interplay Between Natural and Artificial Computation, IWINAC 2017, Corunna, Spain, 19–23 June 2017.

[53] Yang D., Sun J. Proximal dehaze-net: A prior learning-based deep network for single image dehazing. Proceedings of the European Conference on Computer Vision (ECCV).

[54] Zhang J., He F., Chen Y. (2020). A new haze removal approach for sky/river alike scenes based on external and internal clues. Multimed. Tools Appl..

[55] Sun Y., Sun Z., Chen W. (2024). The evolution of object detection methods. Eng. Appl. Artif. Intell..

[56] Liu S., Zeng Z., Ren T., Li F., Zhang H., Yang J., Jiang Q., Li C., Yang J., Su H. (2024). Grounding dino: Marrying dino with grounded pre-training for open-set object detection. Proceedings of the European Conference on Computer Vision.

[57] Liu S., Zeng Z., Ren T., Li F., Zhang H., Yang J., Jiang Q., Li C., Yang J., Su H. (2024). Grit: A generative region-to-text transformer for object understanding. Proceedings of the European Conference on Computer Vision.

[58] Liu H., Jin F., Zeng H., Pu H., Fan B. (2023). Image enhancement guided object detection in visually degraded scenes. IEEE Trans. Neural Netw. Learn. Syst..

[59] Li Y., Li X., Li W., Hou Q., Liu L., Cheng M.M., Yang J. (2024). Sardet-100k: Towards open-source benchmark and toolkit for large-scale sar object detection. arXiv.

[60] Jiang W., Zhang Z., Xiong Q., Yang B. (2024). A survey of object detection based on deep learning. Proceedings of the Fourth International Conference on Advanced Algorithms and Neural Networks (AANN 2024).

